# Pathophysiological mechanisms and therapeutic approaches in obstructive sleep apnea syndrome

**DOI:** 10.1038/s41392-023-01496-3

**Published:** 2023-05-25

**Authors:** Renjun Lv, Xueying Liu, Yue Zhang, Na Dong, Xiao Wang, Yao He, Hongmei Yue, Qingqing Yin

**Affiliations:** 1 The First School of Clinical Medicine, Lanzhou University, Lanzhou, 730000 China; 2 Department of Endocrinology, Shandong Provincial Hospital Affiliated to Shandong First Medical University, Jinan, 250021 China; 3 Department of Geriatrics, the 2nd Medical Center, Chinese PLA General Hospital, Beijing, 100853 China; 4 Department of Pulmonary and Critical Care Medicine, The First Hospital of Lanzhou University, Lanzhou, 730000 China; 5 Department of Geriatric Neurology, Shandong Provincial Hospital Affiliated to Shandong First Medical University, Jinan, 250021 China

**Keywords:** Respiratory tract diseases, Metabolic disorders

## Abstract

Obstructive sleep apnea syndrome (OSAS) is a common breathing disorder in sleep in which the airways narrow or collapse during sleep, causing obstructive sleep apnea. The prevalence of OSAS continues to rise worldwide, particularly in middle-aged and elderly individuals. The mechanism of upper airway collapse is incompletely understood but is associated with several factors, including obesity, craniofacial changes, altered muscle function in the upper airway, pharyngeal neuropathy, and fluid shifts to the neck. The main characteristics of OSAS are recurrent pauses in respiration, which lead to intermittent hypoxia (IH) and hypercapnia, accompanied by blood oxygen desaturation and arousal during sleep, which sharply increases the risk of several diseases. This paper first briefly describes the epidemiology, incidence, and pathophysiological mechanisms of OSAS. Next, the alterations in relevant signaling pathways induced by IH are systematically reviewed and discussed. For example, IH can induce gut microbiota (GM) dysbiosis, impair the intestinal barrier, and alter intestinal metabolites. These mechanisms ultimately lead to secondary oxidative stress, systemic inflammation, and sympathetic activation. We then summarize the effects of IH on disease pathogenesis, including cardiocerebrovascular disorders, neurological disorders, metabolic diseases, cancer, reproductive disorders, and COVID-19. Finally, different therapeutic strategies for OSAS caused by different causes are proposed. Multidisciplinary approaches and shared decision-making are necessary for the successful treatment of OSAS in the future, but more randomized controlled trials are needed for further evaluation to define what treatments are best for specific OSAS patients.

## Introduction

OSAS is a highly prevalent sleep-related breathing disorder characterized by hypopnea and apnea in ventilation. These breathing disturbances cause IH, which leads to blood hypoxemia, hypercapnia, fragmented sleep, recurrent nocturnal arousals, enhanced respiratory effort, and increased sympathetic nerve activity.^1,2^ Epidemiologic studies have documented the incidence of OSAS in the general population aged 30–60 years to be 24% in men and 9% in women,^3,4^ and a recent study reported almost 1 billion affected people globally,^5^ which has aroused extremely important concern (Table 1). Obesity, age, and sex have been identified as risk factors for OSAS, and other risk factors are related to ethnicity, family history, and poor lifestyle habits (alcoholism and smoking).^6,7^ The risk of OSAS correlates with body mass index (BMI), in which OSAS increases progressively with increases in BMI, most likely related to upper airway narrowing due to excess fat tissue.^8^ Obesity can induce a decrease in vital capacity, an imbalance in the ventilation-perfusion ratio, and limitations of lung and chest wall movement.^8^ As a result of this association, the countries with the highest incidence of OSAS are those with high rates of obesity, and thus, the incidence of OSAS increases with increasing levels of obesity.^9^ OSAS can occur at all ages, the incidence of OSAS has a tendency to increase with age, and the number of apnea events occurring during the night is usually higher in healthy older people than in middle-aged adults, reaching a plateau after approximately 65 years of age.^8,10,11^ Male sex is an independent risk factor for OSAS, with a male predominance and an estimated male-to-female prevalence of 1.5:1,^12^ and the reasons for this disparity are incompletely understood. The prevalence of OSAS increases in postmenopausal women, probably because body fat is redistributed to the upper body.^13,14^ In addition, the protective effects of female hormones, such as progesterone and estrogen, are decreased in the postmenopausal period.^15^ Symptoms of OSAS appear nonspecific and include snoring, apnea, arousal, and daytime sleepiness. Table 2 shows that day and night can be distinguished with respect to the major signs and symptoms of OSAS.^1,16^ According to current international recommendations, the diagnosis of OSAS is made after a sleep examination, and polysomnography (PSG) monitoring is applied as a method to diagnose OSAS, with the application of the 2017 scoring rules.^17^ These rules define apnea as a 90% reduction in airflow that lasts at least 10 s. Hypoventilation is defined as a decrease in flow of at least 50% and a decrease in oxygen saturation of 3% for at least 10 s. The severity of OSAS is distinguished clinically by the number of apnea–hypopneas per hour of sleep and the apnea-hypopnea index (AHI). AHI <5 is defined as no sleep apnea, AHI 5–15 as mild OSAS, AHI 15–30 as moderate OSAS, and AHI >30 as severe OSAS, and sleep apnea events identified in the sleep record of individuals without any symptoms are not considered OSAS unless AHI >15.^17,18^

**Table 1:** Incidences of apnea and hypopnea frequencies in various parts of the world

Country/Region	Study population	Year(s) of data collection	Age range (years)	Scoring criteria	AHI ≥5		AHI ≥15		Reference
					Men	Women	Men	Women	
USA	1520 adult employed individuals in the Wisconsin Sleep Cohort Study	1988–2011	30–70	AASM 2007	33.9%	17.4%	13.0%	5.6%	Peppard et al. (2013)^9^
USA	5,804 participants in the Sleep Heart Health Study Cohort	1995–2006	å 40	AASM 2012	32.4%	25.3%	26%	12.3%	Donovan et al. (2016)^664^
Hong Kong	153 male office-based workers in Hong Kong	1997–1999	30–60	AASM 2007	8.8%	-	5.3%	-	Mary et al. (2001)^665^
Hong Kong	106 female office staff members of public institutions in Hong Kong	1998–2000	30–60	AASM 2007	-	3.7%	-	1.9%	Mary et al. (2004)^666^
Australia	380 residents of the rural town of Busselton in the state of Western Australia who were participants in the Busselton Health Study	1990	40–65	AASM 2012	25.5%	23.5%	4.7%	4.9%	Marshall et al. (2008)^667^
Japan	322 male employees of a wholesale company	2004–2005	23–59	AASM 2012	59.7%	-	22.3%	-	Yukiyo et al. (2008)^668^
Singapore	242 individuals in the Singapore Health Study 2012	2012	21–79	AASM 2012	70.8%	70.8%	30.5%	30.5%	Adeline et al. (2016)^669^
Switzerland	2121 participants in the HypnoLaus Sleep Cohort study	2009–2013	40–85	AASM 2012	83.8%	60.8%	49.7%	23.4%	Heinzer et al. (2015)^670^
Russia	1050 participants in the ARKH sleep study	2014–2018	30–70	AASM 2017	14.1%	19.5%	3.7%	5.9%	Anna et al. (2020)^671^
Brazil	1042 volunteers in the Sao Paulo Epidemiologic Sleep Study	2008	20–80	AASM 2007	46.5%	30.6%	24.8%	9.6%	Sergio et al. (2010)^672^
Germany	1208 persons who participated in SHIP‐Trend	2008–2012	20–81	AASM 2007	59%	33%	30%	13%	Ingo et al. (2019)^673^
Iceland	415 subjects in the European Community Respiratory Health Survey	2012–2013	40–65	AASM 2007	13.3%	10.8%	10.6%	4.8%	Arnardottir et al. (2016)^674^
New Zealand	364 Māori and non-Māori New Zealanders	1999–2001	30–59	AASM 2007	12.5%	3.4%	3.9%	0.2%	Mihaere et al. (2009)^675^
Norway	518 subjects in the Akershus Sleep Apnea Project	2006–2008	30–65	AASM 2007	21%	13%	11%	6%	Harald et al. (2011)^676^
Spain	2148 subjects from Vitoria-Gasteiz, Basque Country (Spain)	1993–1997	30–70	AASM 2007	26.2%	28%	14.2%	7%	Durán et al. (2001)^677^
South Korea	457 participants of a study that included residents of Ansan community (Southwest Seoul)	2001	40–69	AASM 2007	21.7%	16.8%	10.1%	4.7%	Kim et al. (2004)^678^
Poland	676 adult inhabitants of Warsaw in the MONICA II study	1993	41–72	AASM 2007	36.2%	18.4%	15.8%	7.6%	Robert et al. (2008)^679^
India	365 subjects from the South Delhi district	2005–2007	30–65	Chicago 1999	13.5%	6.1%	5.5%	6.1%	Reddy et al. (2009)^680^
China	309 patients with type 2 diabetes mellitus in Beijing	2016–2017	40–70	AASM 2012	68.3%	62.4%	38%	30.7%	Ding et al. (2022)^681^
Chile	205 Chilean adults enrolled in the 2016/17 National Health Survey	2016–2017	18–84	AASM 2007	62%	31%	21%	13%	Fernando et al. (2020)^682^
Canada	215 individuals in the First Nations Sleep Health Project	2018–2019	18–76	AASM 2017	51.1%	41.7%	14.8%	9.4%	James et al. (2022)^683^

We searched PubMed, Embase, the Cochrane Library, and ClinicalTrials.gov. Finally, high-quality and representative studies from 19 countries or regions were included

*AASM* American Academy of Sleep Medicine, *AHI* apnea-hypopnea index

**Table 2:** Day and night can be distinguished with respect to the major signs and symptoms of obstructive sleep apnea syndrome (OSAS)

**A. Nocturnal symptoms**
Snoring and observed apnea are the most frequent and hallmark nocturnal symptoms of OSAS, both of which reflect the critical narrowing of the upper airway. Nocturnal asphyxia also appears to be helpful in identifying patients with OSAS
a. Snoring: Snoring is the most characteristic nocturnal symptom of OSAS; patients with OSAS tend to have a long-standing history of snoring, which becomes increasingly intense and irregular over time
b. Observed apneas: Apneas are a frequent cause of consultation, since they often cause concern for the partner of the patient, describing them as respiratory pauses that interrupt snoring while the patient continues to struggle to breathe. Apnea alternates with snoring, and apneas occur after cessation of snoring, accounting for ~40% of sleep time
c. Arousals: Patients may experience arousal or distress when they experience apnea, feelings of terror, hand swings, or body movements. Arousals are less frequent than observed apneas. This symptom is associated with hypertension, since recurrent arousals are related to sympathetic discharges that elevate blood pressure and heart rate
d. Other: Night sweats, nocturia, restless sleep, somniloquy, and symptoms of gastroesophageal reflux are additional nocturnal symptoms related to OSAS
**B. Daytime symptoms**
a. Daytime sleepiness: Most patients have significant excessive daytime sleepiness (EDS), poor concentration and tiredness, which is due to sleep fragmentation. In addition, morning distension or headache, apathy, depression, irritability and/or changes in affect, memory loss, social issues, decreased libido, and erectile dysfunction are other characteristic daytime symptoms of patients with OSAS

Over the past decades, research progress on the pathophysiology of OSAS has been relatively slow due to the limitations of disease models. Reviewing previous studies, we showed that IH can induce alterations in multiple signal transduction pathways that could affect various systems and organs throughout the body. Epidemiological studies have reported a positive association between OSAS and increased risks of cardiovascular diseases,^19,20^ neurological disorders,^21,22^ and metabolic diseases.^23,24^ Additionally, a number of studies have shown that OSAS plays a crucial role in the development of nonalcoholic fatty liver disease.^25–27^ Recently, increasing evidence from our laboratories and others has shown that OSAS is also involved in a number of other diseases, including insulin resistance,^28,29^ glucose metabolism,^30^ kidney disease,^31^ hypertension,^32,33^ cancer,^1,34^ the immune system,^35^ and gastroesophageal reflux.^36^ However, the pathogenic mechanisms of OSAS in organs are complex and intertwined and not fully understood. In this review, the pathophysiological mechanism of OSAS and the relationship between the alterations in potential signaling pathways and multiple systemic diseases are described in detail and comprehensively, and the corresponding therapeutic strategies for different pathogeneses are discussed.

## Mechanisms/pathophysiology of OSAS

**Fig. 1:**
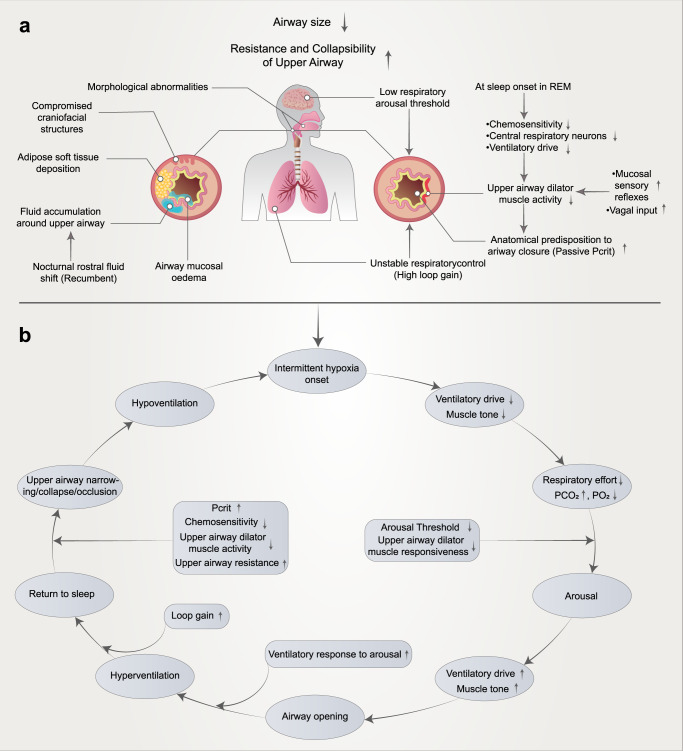
Mechanisms influencing upper airway collapse in the pathogenesis of OSAS (**a**) and the interplay between various factors (**b**). The reduction in upper airway volume caused by obesity or craniofacial structural abnormalities and soft tissue changes is an important factor in upper airway collapse. All OSAS patients have different degrees of upper airway anatomical structure injury. A nocturnal rostral fluid shift is defined as fluid accumulated in the legs during the daytime, redistributing to the upper part of the body upon lying down at night, causing an increase in peripheral pressure. In addition, most patients have mucosal edema, and the mechanism is not clear. Furthermore, several mechanisms associated with a low respiratory arousal threshold, poor pharyngeal neuromuscular muscle responsiveness, high loop gain, and high passive Pcrit may involve OSAS. When awake, neuronal activity ensures that the muscles of the dilated throat are activated, thereby preventing collapse. When this muscle loses activation during rapid eye movement (REM) sleep (chemosensitivity, central respiratory neurons, and ventilatory drive), the airway may collapse. Schematic representation of multiple pathological factors interacting to promote cyclical OSAS pathogenesis (**b**). In addition, these mechanisms might represent therapeutic targets. In the treatment section of this article, we introduce targeted therapies for different mechanisms

The pathophysiological mechanisms underlying OSAS are complex and multifactorial, and furthermore, the underlying causes of OSAS vary substantially between afflicted individuals, with many unknown and poorly understood aspects. With the increase in OSAS-related research, it is gradually recognized that there are anatomical factors and functional factors involved in the mechanism of upper airway collapse. Based on the involvement of anatomical and nonanatomical factors in the pathogenesis of OSAS, a model of PALM pathogenesis was proposed,^37^ which can be summarized as pharyngeal critical closing pressure (Pcrit, P), decreased respiratory arousal threshold (arousal threshold, A), increased loop gain (loop gain, L), and upper airway dilator muscle activity (muscle responsiveness, M). (Fig. 1a). Various pathophysiological factors interact to contribute to the pathogenesis of OSAS (Fig. 1b). The following sections will focus on reviewing the key pathophysiological factors of OSAS and their interactions to highlight innovations in our understanding of OSAS pathogenesis.

### Upper airway collapse

Upper airway anatomical abnormalities are a key factor in the pathogenesis of OSAS. Almost all patients have upper airway anatomical abnormalities to varying degrees, that is, upper airway stenosis and collapse caused by abnormal bone structure and soft-tissue hyperplasia. Upper airway anatomical abnormalities include relative stenosis due to fat deposition in the upper airway caused by obesity and absolute stenosis due to abnormalities in the maxillofacial structure, which are important causes of upper airway collapse.^38^ In addition, patients with leg edema due to cardiac and renal failure or venous insufficiency may experience a shift in leg fluid volume from the leg to the neck during the night, which may lead to upper airway collapse.^39^ Interestingly, the degree of collapse of a particular airway can be measured by calculating the Pcrit (see below for more details).

#### Morphological abnormalities

Morphological abnormalities are the most common contributing factor to the development of OSAS, and in adult patients with OSAS, a reduced mandibular body length, inferiorly positioned hyoid bone, posterior displacement of the maxilla, and narrowing of the pharyngeal space all result in oral cavity crowding.^40–42^ Abnormalities in anatomical features, conditioned by skeletal abnormalities as in Pfeiffer syndromes (craniofacial synostosis) or Pierre Robin syndrome (midface hypoplasia) and Crouzon syndromes and Apert syndromes are also implicated in OSAS.^43^

Enlargement of soft-tissue structures in and around the airways is an important cause of pharyngeal airway narrowing in most cases of OSAS. Examples include excessive or elongated tissues of the soft palate, retrognathia, macroglossia, enlarged tonsils, increased soft tissue in the neck, and a redundant pharyngeal mucosa.^16^ The enlarged soft palate and tongue invade the airway diameter in the anteroposterior plane, whereas the thickened pharyngeal wall invades the lateral plane,^44^ a major site of airway narrowing in most patients with OSAS.^45^ Obesity rates are high in patients with OSAS. Obesity is a major factor contributing to the compression of the respiratory tract through an increase in the area and volume of fat deposition in the pharynx, and fat deposition in the upper airways and around the thoracic cavity may promote the development of OSAS.^45,46^ In addition, tongue shape might play an important role in the development of OSAS, and studies have found that the tongue shape in patients with OSAS is different from that in normal subjects in the supine position.^47^

#### Nocturnal rostral fluid shift

Fluid retention may contribute to the pathogenesis of OSAS, and nocturnal rostral fluid shift refers to the nighttime redistribution of fluid accumulated in the legs to the upper parts of the body while lying in bed.^48^ The passive movement of isotonic fluid between capillaries and the interstitial space is determined by capillary hydrostatic versus colloid osmotic pressure.^49^ When moving from the recumbent to the upright position, the hydrostatic pressure in the leg capillaries (90–120 cmH_2_O) exceeds the hydrostatic pressure in the interstitial space (15–20 cmH_2_O) due to gravity, thus causing fluid to seep from the capillaries into the interstitial space.^50–52^ Thus, while standing, the plasma volume is reduced by 300–400 ml due to venous pooling and fluid infiltration into the interstitial space, but the leg volume is increased by 100–300 ml.^39^ Fluid that accumulates in the interstitial space enters the circulation through the lymphatic system to maintain a stable interstitial volume. Once the lymphatic excreting capacity is saturated, the fluid accumulated in the interstitium is proportional to the standing time, and the gradient from the foot to the heart decreases.^53^ Upon lying down, the lower limb blood volume is rapidly reduced, and fluid is redistributed to the chest and neck.^54,55^ In addition, when lower body positive pressure was applied to the leg, the fluid was removed from the leg, and the neck circumference increased within 1 min, indicating that the fluid was able to move quickly to the neck.^56–58^ In summary, daytime postures, such as prolonged sitting or standing, causes fluid to accumulate in the intravascular and interstitial spaces distal to the lower extremities. During recumbency, patients may experience a shift in leg fluid capacity from the legs to the neck, increasing tissue pressure and resulting in narrowing of the upper airway, which increases its collapsibility and predisposes them to OSAS.^45,46^ It has recently been documented that the accumulation of even a relatively small amount (100–200 ml) of edema fluid expands the upper airway soft-tissue structures in patients with OSAS and snorers.^59^ Changes in leg circumference at night have been shown to correlate strongly with changes in neck circumference and AHI.^39^

#### Passive airway collapsibility

Although upper airway obstruction may be due to a variety of factors, such as obesity, there is increasing evidence that individual collapsibility is also a key factor in upper airway obstruction.^60–62^ The importance of abnormal pharyngeal susceptibility to collapse in the pathogenesis of obstructive apnea was demonstrated by studying the Pcrit in patients with OSAS and in control subjects.^63^ A highly collapsed upper airway is the leading cause of OSAS pathogenesis, and the passive Pcrit technique is considered the gold standard for measuring the degree of pharyngeal airway collapse.^64^ The Pcrit is the pressure at which the airway fails to remain open and collapses,^61,65^ and previous investigators have demonstrated that in normal individuals, Pcrit is negative,^66^ implying that the upper respiratory airway tends to remain open. In patients with OSAS, the critical pressure is less negative, which means that the upper respiratory airway is more likely to collapse and become occluded during sleep.^66,67^ Applying a theoretical model of upper airway obstruction, researchers could represent the upper airway as a simple tube with collapsible parts. Any increase in pressure around the tube will exceed the internal pressure in the tube, causing pharyngeal collapse. When the pressure around the tube increases to the level of the pressure inside the tube, it is called the Pcrit of that segment.^64^ Therefore, the pharyngeal critical closing pressure refers to the pressure acting on the upper airway. In the absence of muscle activity, the pharynx will close, and it could reflect the mechanical properties or collapsibility of the pharynx. The more negative the tube pressure, the less effort is required to open the airway compared to atmospheric pressure. A growing body of literature has shown that Pcrit is higher in patients with greater upper airway collapsibility. The critical closing pressure of the airway was higher in patients with OSAS than in those without the disorder.^68,69^ Pcrit is a vital part of categorizing subjects with OSAS into various endotype groups, which could provide help for the treatment and response prediction of OSAS patients.^70^

### Decreased respiratory arousal threshold

In recent years, a number of studies have shown that a low respiratory arousal threshold may be an important endotype of OSAS.^71–73^ Each OSAS event terminates with brief brain activation in a process called arousal or microarousal.^1^ The tendency of OSAS patients to wake easily during sleep-disordered breathing is called the low arousal threshold. The arousal threshold varies between individuals,^74^ and studies have found that at least one-third of OSAS patients have a decreased respiratory arousal threshold.^75^ Arousal plays a dual role in the mechanism of OSAS. On the one hand, arousal from sleep at the end of a respiratory event is an important protective mechanism for restoring pharyngeal patency,^76^ and patients will resume normal breathing and relieve airway obstruction through neuromuscular and respiratory compensation mechanisms during arousal.^77^ Thus, respiratory arousal is considered a potentially lifesaving event that could avert asphyxia during sleep. On the other hand, a decreased respiratory arousal threshold is the cause of recurrent microarousal in OSAS patients. Recent studies also suggest that frequent arousals might lead to the interruption of sleep continuity, prevent deeper and more stable sleep, reduce the ability to recruit upper airway dilator muscles, and may contribute to further obstructive respiratory events.^72,76–78^ Arousal intensity is a unique pathophysiological phenotype, and individuals with a more intense arousal tendency to airway stenosis elicit a greater ventilatory response and are, therefore, more likely to experience instability in ventilatory control.^79^ Theoretically, hyperventilation during arousal would also reduce pharyngeal muscle activity,^76,77^ and in many cases, arousal might promote the cyclical breathing pattern of OSAS.^78^ Experimentally, the respiratory arousal threshold is measured by the lowest pressure in the esophagus produced during a respiratory event or perturbation of a breath taken before awakening. Evidence suggests that the magnitude of the intrapleural pressure generated by breathing is a major stimulus for the initiation of arousal from sleep.^80–82^ Although arousal thresholds vary widely between individuals, patients with OSAS tend to have diminished arousal responses to airway obstruction compared with controls, which may exacerbate upper airway dilator hypotonia, leading to an inability to recruit dilator muscles to open the airway before arousal occurs.^46,79^

### Increased loop gain

In ventilatory control, loop gain is a measure of respiratory instability, which refers to unstable ventilatory chemoreflex control and is recognized as a key pathophysiological feature that contributes to OSAS.^83–85^ Eckert’s study has shown that approximately 36% of OSAS patients have high loop gain.^37^ The loop gain consists of the control gain, plant gain, and cycle time.^86^ Control gain refers to the response degree of the respiratory system to the change in PaCO_2_, plant gain is characterized by the efficiency of the respiratory system in responding to the reduction in CO_2_ by ventilation, and cycle time refers to the feedback time from the change in PaCO_2_ and PaO_2_ in blood being received by the sensor to the ventilatory response of the body.^87^ High control gain represents a strong chemoreceptor response to a small change in PaCO_2_, and high plant gain indicates that a mild ventilatory response can cause a significant change in PaCO_2_.^88^ For example, upper airway muscles are innervated by neuronal fibers from the respiratory center, high ventilation caused by high loop gain can expel more CO_2_, and low serum CO_2_ levels reduce the central ventilatory drive in the dilator muscles of the upper airway, thereby reducing pharyngeal muscle activity.^89,90^ Thus, the higher the loop gain is, the less stable the ventilatory chemoreflex control. Unstable ventilatory chemoreflex control could promote airway collapse in OSAS due to hypocapnic (produced by hyperventilation after obstructive apnea) hypotonia of the upper airways. Obstructive apnea is followed by hyperventilation, producing hypocapnia and respiratory depression, which contribute to the instability of ventilatory chemoreflex control and high loop gain,^1,46,83,91^ and increased CO_2_ from hypoventilation leads to the development of rapid and large negative inspiratory pressure, also leading to a collapse of the upper airway. In addition, high loop gain could lead to a mismatch between the driving force of the respiratory center on the respiratory muscles and the driving force of the upper airway dilator muscles; that is, the activity of the upper airway dilator muscles is not sufficient to counter the negative suction generated by the respiratory muscles during inspiration, which leads to upper airway stenosis and collapse.^89,90^

### Decreased upper airway dilator muscle activity during sleep and impaired sympathetic neural activity

Increased pharyngeal dilator muscle activity in OSAS patients compared with matched controls has been interpreted as evidence of a neuromuscular protective compensatory reflex in response to anatomical compromise in OSAS.^80^ When awake, neuronal activation of the dilator muscles ensures that the pharyngeal dilator muscles are activated, thus preventing pharyngeal narrowing and collapse and protecting pharyngeal patency. When this upper airway dilator muscle activation is lost at the onset of sleep, its ability to maintain a patent airway decreases, and in turn, the airway could narrow and/or collapse.^1,45^ The genioglossus muscle is the most important pharyngeal dilator and has pharyngeal mechanoreceptors and chemoreceptors that deliver the relevant stimulus signals received (carbon dioxide in the blood) to the brainstem, tuning the upper airway dilator activity. Impairments in this process may lead to a reduction in the expansion forces of the pharyngeal dilator muscles, and the reduced pharyngeal caliber increases the likelihood of an obstructive event, in addition to the incoordination between the inspiratory activity of the muscles and the respiratory effort, increasing the resistance of the upper airway.^16,45,80,92,93^

### Mechanisms of central sleep apnea

Central sleep apnea (CSA) is a sleep-breathing disorder characterized by apnea and hypopnea caused by a lack of drive to breathe during sleep.^94^ The occurrence of respiratory events can be intermittent or periodic, and patients could also experience obstructive respiratory events. In contrast, OSAS is apnea or hypopnea due to repeated collapse or obstruction of the upper airway during sleep, which is characterized by the weakening or disappearance of oronasal airflow, while chest and abdominal motion or respiratory effort is still present.^89^ CSA is not as common as OSAS in clinical practice and accounts for less than 10% of all sleep-related breathing disorders,^95^ so it has received less attention. Similar to OSAS, CSA is associated with important complications, including frequent night awakenings, excessive daytime sleepiness, and an increased risk of adverse cardiovascular outcomes,^96^ and CSA has been divided into eight categories by the International Classification of Sleep Disorders, Third Edition (ICSD-3).^18^ Table 3 summarizes the differences between OSAS and CSA. Neurophysiologically, CSA is due to a temporary failure of the pontomedullary pacemaker to generate breathing rhythm. Thus, without brainstem inspiratory nerve output, the nerves innervating all inspiratory muscle groups are silent, which results in a loss of inspiratory ventilatory effort.^96,97^ Although the exact pathogenesis of different types of CSA might vary considerably, unstable ventilatory drive during sleep is the main characteristic. Sleep phases can be divided into nonrapid eye movement (NREM) sleep, rapid eye movement (REM) sleep, and wakefulness. CSA and instability in humans mainly occur in NREM sleep, and the mechanism is related to the high loop gain in NREM sleep.^88,98,99^ Under the joint action of high control gain and high plant gain, the sensitivity of the ventilation control system would be increased, but only two points cannot cause the occurrence of CSA. There must be a certain time interval between the effect produced by the effector (lung) (increase or decrease in ventilation) and the change in CO_2_ sensed by the receptor (peripheral or central chemoreceptors), which is the key to the eventual onset of apnea.^89^ Under the action of some factors, the increased PaCO_2_ will act on the peripheral chemoreceptors and cause a ventilatory response, which will lead to a decrease in PaCO_2_. Under normal circumstances, PaCO_2_ will finally reach the dynamic equilibrium state. Interestingly, elevated PaCO_2_ is rapidly corrected in patients with CSA, and the initiating factor driving the ventilatory response may have normalized, while due to delayed signal cycling caused by a prolonged cycle time, this signal is not promptly fed back by the receptor to the effector, at which point the effector is still performing ventilatory commands and finally results in hyperventilation.^100^ If PCO_2_ falls below the chemoreceptor detection threshold, the respiratory drive is eliminated, and CSA occurs.^101–103^ In the event of CSA, the oscillatory cycle that leads to the recurrence of CSA is perpetuated by the following factors: pharyngeal stenosis requiring sufficient expansion tension to overcome gravity and tissue adhesion and inconsistencies between normal and actual PCO_2_ levels at which respiratory rhythm resumes following CSA.^104–106^ Compared with OSAS, although a large number of studies have been conducted in the past 20 years, the etiology and pathophysiological mechanism of CSA are complex, so the understanding of CSA is still insufficient and needs to be further explored and improved.

**Table 3:** Differences between obstructive sleep apnea syndrome (OSAS) and central sleep apnea (CSA)

	OSAS	CSA
Definition	OSAS is a sleep-related breathing disorder associated with an obstruction in the upper airway that results in an increased breathing effort and inadequate ventilation.	CSA is defined by the recurrent cessation of respiration during sleep not associated with ventilatory effort
Prevalence	The incidence of OSAS was 24% in men and 9% in women aged 30–60 years	It accounts for less than 10% of all sleep-related breathing disorders
Common etiology	Obesity; advanced age; male sex; genetic predisposition; menopausal, postmenopausal; upper airway disease. Other associated diseases: hypothyroidism, acromegaly, hypopituitarism, amyloidosis, vocal cord paralysis, sequelae of polio or other neuromuscular disorders (Parkinson’s disease), long-standing gastroesophageal reflux	Neuropathy: nervous system tumors, trauma, angioembolism, intracranial infection; dysautonomia: familial dysautonomia, Shy-Drager syndrome; myopathy: diaphragmatic myopathy, myotonic dystrophy occipital foramen magnum developmental malformation, lateral medullary syndrome. Others: congestive heart failure, nasal obstruction, OSAS after tracheotomy or uvulopalatopharyngoplasty
Pathogenesis	After patients with OSAS fall asleep, the central respiration drive is reduced, and the activity of the pharyngeal dilator muscles is diminished, which, combined with defects in airway anatomy, increases upper airway resistance; the balance of forces to maintain airway opening and closing is thus broken, and the airways collapse, with apnea occurring (see text for details)	When transferring from wakefulness to sleep, the responsiveness of the respiratory centers to various stimuli (e.g., high PaCO_2_ versus low PaO_2_ and pulmonary and respiratory resistive loads) is reduced, i.e., the threshold for responsiveness is elevated; instability of the central nervous system to respiratory feedback control induced by pathological states such as PaCO_2_ and hypoxia
Clinical manifestations	Common in obese patients; increased daytime sleep; the number of awakenings during sleep is minimal; strong snoring; cognitive decline; morning headache; nocturnal enuresis	Normal weight; insomnia is common, but somnolence is rare; more arousals during sleep; snoring is light and intermittent; depressive symptoms; decreased libido

## Intermittent hypoxic injury induced by OSAS: alterations in signaling pathways

### The role of HIF-1α under different oxygen conditions

**Fig. 2:**
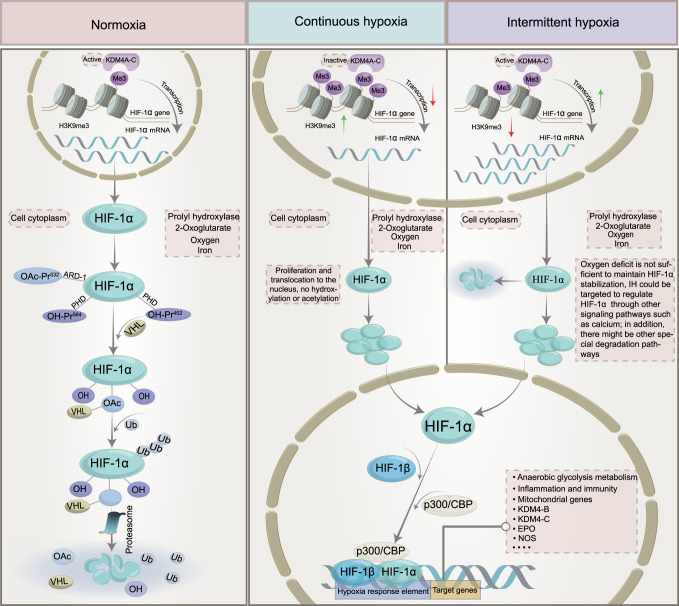
The mechanism of HIF‐1α activation and degradation under intermittent and continuous hypoxia conditions. Under normoxic conditions, HIF-1α is transcribed in the nucleus and translated into HIF-1α protein in the cytoplasm, which is normally hydroxylated by PHD. It then interacts with the VHL protein, undergoes ubiquitination, and is destroyed. Under continuous hypoxia, HIF-1α does not degrade but translocates to the nucleus, where it binds with HIF-1β and then recruits p300/CBP on HRE to initiate gene transcription. Among them, the HIF-1α target genes KDM4B and KDM4C were upregulated. Despite the elevated enzyme levels of KDM4A, KDM4B, and KDM4C, KDM activity was not maintained by the limited amount of oxygen, and KDM4A, KDM4B, and KDM4C remained largely inactive. This leads to increased H3K9me3, which ultimately reduces the amount of HIF-1α mRNA transcribed. Under intermittent hypoxia conditions, HIF‐1α was partially degraded during the reoxygenation phase, but the levels of KDM4B and KDM4C were increased but not to the level of continuous hypoxia. However, in contrast to continuous hypoxia, KDM4A, KDM4B, and KDM4C showed increased activity, resulting in higher H3K9me3 demethylation of the HIF‐1α gene than in normoxia or continuous hypoxia. This leads to increased production of HIF‐1α mRNA. KDMs histone lysine demethylases, H3K9me3 histone 3 lysine 9 trimethylation, HIF‐1α hypoxia-inducible factor-1, OAc acetoxy, OH hydroxyl, PHD prolyl hydroxylases, VHL von Hippel‒Lindau, EPO erythropoietin, NOS nitric oxide synthase, CBP coactivator-binding protein, HRE hypoxia response element

Due to the importance of oxygen for cell survival, metazoans have evolved mechanisms to sense changes in oxygen levels in the cellular microenvironment and trigger adaptive responses during evolution. It is increasingly recognized that the adaptation of organisms to hypoxia depends on the activation of specific oxygen-sensitive genes.^107–109^ A variety of redox-sensitive transcription factors have been identified, with the key factors being the HIF (hypoxia-inducible factor) family (including HIF-1, HIF-2, and HIF-3).^110,111^ HIF-1 is ubiquitously expressed in various tissues, whereas HIF-2 shows a tissue-specific expression pattern and is mainly expressed in a variety of immune cell subtypes, such as macrophages, neutrophils, and lymphocytes.^112–115^ The expression and role of HIF-3 in some immune cells remain unclear. These transcriptional regulators respond to fluctuations in oxygen levels and bind to specific DNA sequences to induce or repress genes, ultimately initiating adaptive transcriptional responses.^116^ Chief among these is HIF-1, which is a dimer consisting of the HIF-1α and HIF-1β subunits.^117^ The expression of HIF-1α is regulated at the level of transcription and translation, and multiple factors regulate the stability and activity of HIF-1α in oxygen-dependent or oxygen-independent ways at the posttranslational level.^118,119^ Under sufficient oxygen conditions, the oxygen sensitivity of the HIF-1α pathway is controlled by prolyl hydroxylase (PHD).^120^ The hydroxylase induces the hydroxylation of HIF-1α proline residues (Pro402 and Pro564) in the presence of oxygen, 2-oxoglutarate, and iron.^121,122^ Moreover, acetylation of HIF-1α at Lys532 by arrest-defective-1 (ARD-1) contributes to the reaction of HIF-1α with the von Hippel-Lindau (VHL) protein,^123^ followed by ubiquitylation of the alpha subunit of HIF-1 and finally ubiquitin-tagged HIF-1α protein degradation by the 26S proteasome^124–126^ (Fig. 2). During hypoxia, the oxygen required for HIF-1α ubiquitination is lost, and the enzyme activity associated with hydroxylation is weakened. Thus, HIF-1α escapes degradation, moves to the nucleus to bind to HIF-1β,^127^ and recruits the transcriptional coactivator (CREB)-binding protein (CBP) and p300^128,129^ to the HIF-1α binding site with hypoxia response elements (HREs)^130,131^ (Fig. 2). The result is the upregulation of a large number of target genes that promote hypoxia adaptation, and over 100 HIF-1α target genes have been identified thus far.^132,133^ These genes are involved in various biological processes, including anaerobic glycolysis metabolism,^134,135^ inflammation and immunity,^115,136,137^ erythropoiesis,^138,139^ metabolism,^140^ angiogenesis,^141,142^ cell survival and apoptosis,^143,144^ and cancer metastasis.^145^ In addition, the downregulation of some genes, such as PDK1, resulted in decreased mitochondrial oxygen consumption.^146^

Similar to chronic hypoxia (Fig. 2), the essence of intermittent hypoxia is the switching between normoxic and hypoxic states [intermittent hypoxia switching (IHS)], which leads to changes in cellular and molecular functions that are different from chronic hypoxia. Studies have found that prolonged IH (hours to days) increases HIF‐1α activity.^147,148^ However, the molecular mechanisms driving cell behavior in IH compared to chronic hypoxia are less well understood. For example, proline hydroxylation and subsequent ubiquitination pathways are critical for HIF-1α stabilization in continuous hypoxia, and whether they also play a role in IH requires further study. Furthermore, in IH mode, we speculate that the free oxygen deficit is not sufficient to maintain HIF-1α stabilization, but studies on cell culture models of IH have shown that IH can evoke transcriptional activation more than continuous hypoxia for a given duration and intensity.^149,150^ Interestingly, HIF-1α protein levels were found to be lower in HCT116 cells treated with IH than in those treated with chronic hypoxia but were still higher than in normoxia.^151^ When the proteasome inhibitor MG262 was added, the accumulation of HIF-1α was much higher than that observed under chronic hypoxia, indicating that proteasomal degradation occurs at a higher level under IH than under chronic hypoxia,^151^ suggesting that there is another mechanism for HIF-1α degradation under IH conditions. In an experiment with cells cultured in IH, PC12 (pheochromocytoma-12) cells were exposed to alternating cycles of hypoxia and reoxygenation, with one cycle of 1.5% oxygen for 30 s and 20% oxygen for 4 min, to investigate the activation of HIF-1α by IH.^152^ HIF-1α protein and transcriptional activity increased in a stimulation-dependent manner as IH increased from 10 to 30 to 60 cycles.^149^ Interestingly, when cells were subjected to continuous hypoxia for 60 min, equivalent to 120 episodes of IH (30 s each episode), continuous hypoxia for 60 min did not increase HIF-1α protein expression or transcriptional activity.^149^ However, prolonged hypoxia in experiments increased HIF-1α protein expression and transcriptional activity.^149,150^ These observations suggest that IH activates HIF-1α more rapidly than continuous hypoxia. Based on current studies, it has been found that there are differences between continuous hypoxia and IH in the kinetics of protein kinase activation, the downstream targets of protein kinases, and the types of activated protein kinases. In addition, molecular responses activated by IH and continuous hypoxia are also different in many pathological conditions. We propose that novel oxygen-sensing mechanisms may exist in organisms that regulate and fine-tune the cellular hypoxic response depending on the duration of hypoxia (Fig. 2) (see below).

### Histones regulate the expression of HIF-1α induced by IH

Multiple studies have shown that exposure to hypoxia could alter the epigenetic landscape at the cellular chromatin level.^153–160^ Similar changes in epigenetic marks (histone modifications,^161–163^ noncoding RNAs,^164,165^ and DNA methylation^166–168^) have been found in developmental and disease states. The number of studies have found increased histone methylation marks in different mammalian cells exposed to severe and continuous hypoxia.^169–171^ Histone methylation affects gene expression by affecting chromatin structure and altering the accessibility of chromatin to transcription factors.^172^ The nucleosome core consists of two H2A/H2B dimers and an H3/H4 tetramer whose protruding long tails can be covalently modified by methylation (me). Generally, histones are methylated only at lysine (K) or arginine residues, but methylation most often occurs at the K residues of H3 and H4 in the histone tails.^172,173^ The state of histone methylation is strongly associated with transcriptional repression or activation, depending on the position of the modified residues and the number of methyl groups.^174^ For example, lysine 4 methylation of H3 (H3K4me2/3), H3K79me2/3 and H3K36me2/3 is associated with active genes, whereas methylation at H3K9 and H3K27 (H3K9me2/3 and H3K27me2/3) correlates with gene repression.^175,176^ Histone methylation involves many chromatin remodeling proteins, including histone lysine demethylases (KDMs), histone methyltransferases, and other histone-modifying enzymes, and KDMs play an important role in the methylation process.^177,178^ Similar to PHD, which regulates HIF-1α degradation, KDMs require 2‐oxoglutarate, Fe, and oxygen as important cofactors for their activity,^179,180^ and another important feature of KDMs is the presence of a Jumanji-C (JmjC) domain. Given the dependence of this enzyme on oxygen for its activities, KDMs can act as molecular oxygen sensors in cells. Interestingly, Batie et al. found that hypoxia can alter chromatin in a range of human cultured cells by directly affecting JmjC-histone demethylase.^170^ The genomic locations of H3K4me3 and H3K36me3 after brief exposure of cells in culture to hypoxia allow assessment of the transcriptional response of cells several hours later. In addition, KDM5A inactivation was also found to mimic hypoxia-induced cellular responses. The above findings suggest that chromatin responds to oxygen fluctuations through the repression of JmjC-histone demethylase.^170^ Another study found that the H3K27 histone demethylase KDM6A is oxygen sensitive, and its deletion results in the same effect as hypoxia, preventing H3K27 demethylation, disrupting cellular differentiation, and reestablishing H3K27 methylation homeostasis in hypoxic cells, which could ameliorate these impairments.^171^ Upregulation of oxygen-dependent KDMs under persistent hypoxia is thought to increase the demethylation of methylated lysine residues. It has been suggested that the upregulation of KDMs is a compensatory mechanism by increasing the levels of these enzymes to compensate for their reduced activity under oxygen-depleted conditions,^153,181,182^ but oxygen-dependent KDM activity may not be elevated due to the scarcity of oxygen content. In addition, the effect of IH on histone methylation has been less studied than that of continuous hypoxia, and the specific regulatory mechanism of histone methylation and the changes in downstream molecules under different oxygen concentrations are also unclear.

Beyer et al. found that when KDM3A and KDM4B were overexpressed in HeLa cells cultured in 0.2% oxygen, the cells were differentially sensitive to hypoxia. Demethylation of H3K9me3 by KDM4B was decreased, whereas KDM3A activity remained unchanged under the same conditions.^181^ This finding implies that the physiological change from normoxia to hypoxia weakens the enzyme activity and additionally reveals a difference in the apparent oxygen sensitivity of the two JmjC-KDMs. Continuous hypoxia induces a decrease in KDM activity, resulting in global hypermethylation of lysine residues in histones, altering the expression of several genes.^178^ KDMs have been observed to be upregulated (at the mRNA level) in response to continuous hypoxia, but thus far, KDMs have not been identified as HIF-1α target genes.^153,169,183^ Recent studies have found that IH increases HIF-1α activity through pathways that are distinct from chronic hypoxia. Martinez et al. exposed different cell types to IH. HIF-1α protein and HIF-1α target gene (KDM4B and KDM4C) expression were increased under both chronic hypoxia and IH relative to normoxia, and the degree of gene expression was related to the dose-dependent effect of hypoxia. The increased expression of HIF-1α protein and known HIF-1α target genes under intermittent hypoxia is a generalized cellular response.^151,184^ Multiple experiments have compared HIF-1α mRNA levels in HCT116 cells, MCF7 cells, and brain (U251), prostate (PC3), and breast (MDA-MB-231) cancer cell lines after normoxic, chronic hypoxia, and IH exposure.^151,184^ Surprisingly, HIF-1α mRNA expression levels were decreased in chronic hypoxia and increased in IH in all cell lines compared to normoxia.^151,184^ The data suggest that HIF-1α expression is controlled differently in IH and chronic hypoxia. Further studies found that H3K9me3 increases in different cell types exposed to chronic hypoxia relative to normoxia^170,185^; however, unlike chronic hypoxia, IH reduced H3K9me3 levels below those observed with normoxia.^151^ Interestingly, H3K9me3 is associated with heterochromatin and gene silencing,^186^ so the global reduction in H3K9me3 induced by IH may lead to increased expression of associated genes.^178,185^ This finding supports the hypothesis that H3K9me3 reduction mediates the IH-induced increase in HIF-1α gene expression (Fig. 2). In parallel, the protein and mRNA expression of KDM4A, KDM4B, and KDM4C was further assessed. The protein levels of KDM4A were found to be unchanged in cells exposed to normoxia, chronic hypoxia or intermittent hypoxia, and the protein levels of KDM4B and KDM4C were significantly increased in chronic hypoxia compared with IH. Given that KDM4A mRNA levels are reduced in chronic hypoxia and do not change in IH compared to normoxia, it is suggested that KDM4A is not an HIF-1α target gene. Interestingly, several studies have found that the degradation of KDM4A in hypoxia is prolonged via an unknown mechanism,^185,187,188^ resulting in higher levels of KDM4A under hypoxic conditions, although KDM4A may be inactive.^180^ Although the enzyme levels of KDM4A, KDM4B, and KDM4C are increased under conditions of constant hypoxia, they may lose their activity due to hypoxia.^151,178^ Compared with continuous hypoxia, there is sufficient oxygenation between hypoxia fluctuations to remain active in IH, resulting in higher H3K9 demethylation levels of the HIF-1α gene than those in normoxia or chronic hypoxia, resulting in increased HIF-1α mRNA production (Fig. 2). Overall, studying the biological response to OSAS-induced IH is difficult because the patterns and types of IH vary widely in vivo, and it remains to be tested whether this response occurs in all forms of IH. Future studies will contribute to further understanding of how novel cellular oxygen sensors react and interact to generate hypoxic responses in IH.

### ROS-dependent Ca^2+^ signaling pathways and IH-induced HIF-1α activation

**Fig. 3:**
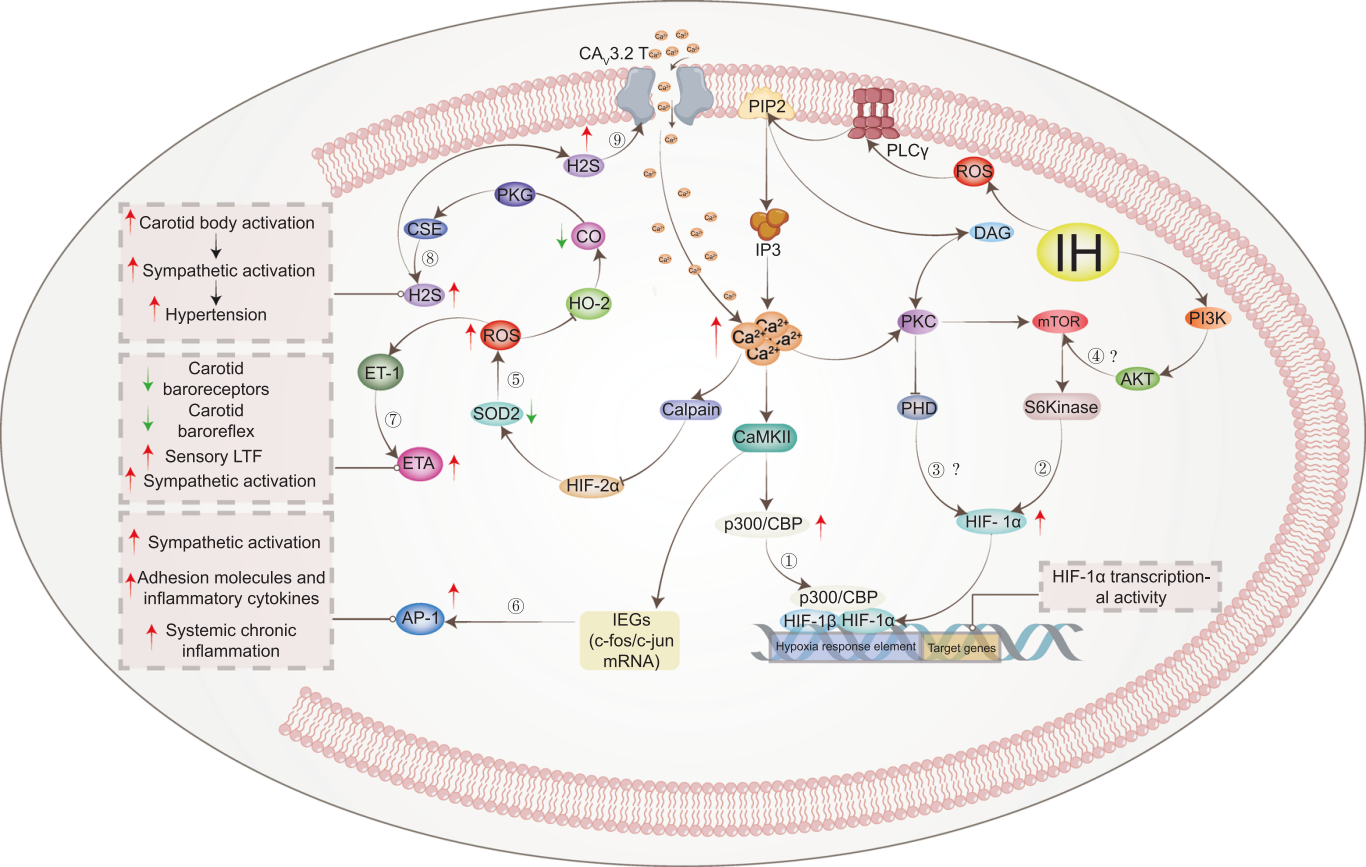
Activation of IH-associated signaling pathways. IH causes an increase in intracellular ROS, which can activate PLC-γ to produce IP3 and DAG. These two messengers are involved in intracellular signal transduction pathways and induce HIF-1α protein expression and transcriptional activity, respectively. Pathway ① indicates that IH-induced transactivation of HIF-1α requires ROS-mediated phosphorylation of the CaMKII-dependent coactivator p300. Pathway ② indicates that hypoxia-induced HIF-1α protein expression is caused by increased synthesis of mTOR, which is dependent on the ROS/Ca^2+^ signaling pathway. However, the mechanism by which PKC inhibits the reduction in PHD and the mechanism of the PI3K/AKT signaling pathway needs to be further confirmed (③④). Pathway ⑤ indicates that calcium-activated calpain promoted the degradation of HIF-2α protein in arterial corpuscles, resulting in a decrease in SOD2 and impaired antioxidant capacity of cells. Pathway ⑥ indicates that CaMKII can activate IEG genes, increase the transcription of c-fox mRNA or c-jun mRNA and increase the expression of AP-1, which is related to the activation of the sympathetic system and systemic inflammation. Pathway ⑦ indicates that increased ROS could stimulate the increased expression of ET and ETA and induce LTF in the carotid body. Pathway ⑧ indicates that IH causes ROS-dependent inhibition of CO production by HO-2, resulting in a decrease in PKG activity and an increase in H2S produced by CSE, which triggers a chemosensory reflex of the carotid body, leading to sympathetic excitation and hypertension. In addition, elevated H2S could activate the CA_V_3.2 T calcium channel on the cell membrane, causing Ca^2+^ influx and further aggravating the damage caused by IH (⑨). IH intermittent hypoxia, PLC-γ phospholipase C γ, PIP2 phosphatidylinositol (4,5) bisphosphate, IP3 inositol-3-phosphate, CaMKII calmodulin-dependent kinase II, IEGs immediate early genes, AP-1 activator protein-1, SOD2 superoxide dismutase 2, ET-1 endothelin 1, ETA endothelin receptor, HO-2 heme oxygenase-2, CO carbon monoxide, PKG: protein kinase G, CSE cystathionine γ-lyase, H2S hydrogen sulfide, LTF long-term facilitation

A number of studies have found that the synthesis and stability of HIF-1α evoked by both IH and continuous hypoxia are closely related to the increase in ROS (reactive oxygen species) produced by NOX activation.^189,190^ Interestingly, increased levels of ROS can activate PLC-γ (phospholipase C γ)^191^ to produce IP3 (inositol-3-phosphate) and diacylglycerol (DAG). Hong et al. found that hydrogen peroxide-induced PLC-γ activation and an IP3 receptor-dependent increase in Ca^2+^ in rat astrocytes.^192^ In addition, Yuan et al. demonstrated that HIF-1α accumulation involved PLC-γ and protein kinase C (PKC) activation in PC12 cells treated with IH. IH-induced transcriptional activation of HIF-1α was blocked by the Ca^2+^ chelator BAPTA-AM or a Ca^2+^/CaMK (calmodulin-dependent kinase) inhibitor, which confirmed the crucial role of the ROS-dependent Ca^2+^ signaling pathway.^149^ A previous study reported that continuous hypoxia resulted in transient (15 min) and moderate (1.5-fold) increases in CaMKII activity, which is an important downstream signaling molecule involved in Ca^2+^-mediated gene regulation, in PC12 cells.^193^ These observations are in sharp contrast to IH, where IH induced an exponential and nearly sixfold increase in CaMKII activity with increasing IH cycles and correlated with increased phosphorylation of the CAMKII protein.^149^ Interestingly, both calmodulin and CaMKII inhibitors prevented IH-induced HIF-1α transcriptional activity but not continuous hypoxia-induced HIF-1α transcriptional activity.^149^ Moreover, CaMKII inhibitors did not effectively inhibit IH-induced HIF-1α protein expression, suggesting that CaMKII-dependent signaling is essential for IH-induced HIF-1α transcriptional activation, while HIF-1α protein expression may be independent of the CaMKII pathway. On the other hand, it was also shown that the signaling pathways associated with HIF-1α activation in response to continuous hypoxia differ significantly from HIF-1α activation in response to IH. Multiple lines of evidence show that p300/CBP proteins^194,195^ are major coactivators of IH-induced HIF-1α transcriptional activation.^196–200^ In a hypoxic PC12 cell experiment, it was found that the IP3 receptor-mediated Ca^2+^ signaling pathway leads to the hyperphosphorylation of p300.^201^ IH increases the transcriptional activity of p300, confirming that CaMKII specifically phosphorylates p300 in vitro, which was blocked by CaMKII inhibitors.^149^ These observations indicate that IH-induced HIF-1α transcriptional activation requires a novel signaling pathway involving CaMKII-dependent activation of p300/CBP coactivators (Fig. 3 ①). Increased Ca^2+^ has been reported to activate classical PKC, which in turn activates mTOR (mammalian target of rapamycin) signaling, a kinase that promotes HIF-1α expression.^202^ Ca^2+^-dependent activation of PKC and mTOR could increase HIF-1α protein expression in PC12 cells.^203^ Interestingly, IH resulted in PKC-dependent mTOR activation compared to continuous hypoxia, and mTOR-dependent increased HIF-1α expression contributed to IH-induced HIF-1α accumulation. At the same time, rapamycin reduced IH-induced HIF-1α stabilization, and IH increased phosphorylated mTOR levels and downstream S6 kinase activation.^190^ In addition, the effects of IH on mTOR activation and HIF-1α protein activity were inhibited by inhibitors of IP3 receptors and PLC-γ as well as the Ca^2+^ chelator BAPTA-AM.^204^ The results further confirmed that IH-induced HIF-1α stabilization was associated with increased protein synthesis and activation of rapamycin-sensitive mTOR signaling (Fig. 3 ②). Similar to the continuous hypoxia report, decreased PHD activity was also found to lead to stable enhancement of HIF-1α after IH, and the negative regulation of PHD activity by PLC-γ/Ca^2+^/PKC/PHD signaling requires further investigation to elucidate the underlying molecular mechanisms (Fig. 3 ③). Based on the present evidence, the Ca^2+^ signaling pathway is involved in IH-induced mTOR activation and subsequent HIF-1α protein accumulation, as well as HIF-1α transcriptional activity. Recent studies have found that hypoxia can activate the PI3K (phosphoinositide 3-kinase)/Akt (protein kinase B) signaling pathway in cells.^205–207^ In addition, the stability of HIF-1α is related to the PI3K/Akt signaling pathway,^206^ and activation of PI3K is required for continuous hypoxia to activate HIF-1α.^208^ Several studies have also found that PI3K inhibitors reduce HIF-1α expression.^206,209,210^ However, neither LY294002 nor wortmannin (two PI3K inhibitors) blocked IH-induced HIF-1α transcriptional activity.^149^ The correlation between the PI3K/Akt signaling pathway and IH is controversial and may be related to the disease and cell type under hypoxic conditions. There are relatively few related studies, and more studies are needed to clarify the relationship between IH and the PI3K/Akt signaling pathway (Fig. 3 ④).

Previous studies have shown that PI3K and mitogen-activated protein kinases (MAPKs) are essential for continuous hypoxia-induced activation of HIF-1α-mediated transcription.^200,211^ In addition, other studies have shown that MAPK inhibitors attenuate hypoxia-induced transcriptional activation of HIF-1α in PC12 cells.^212–214^ Inhibitors of PI3K have also been shown to inhibit HIF-1α protein accumulation and attenuate hypoxia-induced transcriptional activation of HIF-1α.^215^ Although MAPKs (ERK 1/2 kinases; Jun Kinase) could be activated by IH, Yuan et al. examined the effects of MAPKs and PI3K inhibitors on HIF-1α transcriptional activation induced by IH. It was found that neither MAPKs nor PI3K inhibitors prevented HIF-1α transcriptional gene activation induced by IH.^149^ These studies, although preliminary, suggest that IH is associated with transcription factor activation in signaling pathways that are distinct from those used by continuous hypoxia. Another closely related protein, HIF-2α, is processed similarly to HIF-1α and has been reported to be a potent activator of genes encoding antioxidant enzymes.^216^ Several studies have shown that antioxidants such as superoxide dismutase 2 (SOD2) are also downregulated in IH-exposed cells.^217–219^ It has been hypothesized that the downregulation of antioxidants is closely related to HIF-2α downregulation. Interestingly, research has confirmed that IH-induced HIF-2α degradation leads to a significant downregulation of SOD2 transcription, which prevents IH-induced oxidative stress and restores SOD2 activity by ectopic overexpression of transcriptionally active HIF-2α.^218^ Systemic treatment of IH-exposed rats with ALLM (a potent inhibitor of calpains) not only restored HIF-2α in carotid bodies (CBs) and adrenal medulla but, more importantly, restored SOD2 activity and protected against oxidative stress.^218^ The reduction in HIF-2α expression by IH is due to increased degradation of the protein by Ca^2+^-dependent calpain.^218,220^ The degradation of HIF-2α by calpains involves the C-terminus portion of the HIF-2α protein.^117^ In addition, inhibitors of ALLM prevented IH-induced HIF-2α degradation, whereas PHD inhibitors or proteasome inhibitors were ineffective. These observations demonstrate that IH leads to HIF-2α downregulation via Ca^2+^-dependent signaling (Fig. 3 ⑤).

### ROS-dependent Ca^2+^ signaling pathways and IH-induced IEG activation

In the family of proto-oncogenes, there is a class that can be induced by second messengers. These genes are called immediate early genes (IEGs), also known as rapid response genes. The IEG family mainly includes the fos, jun, and myc families.^221^ At present, the c-fos and c-jun families are the most deeply studied. The c-fos gene is one of the most important members of the IEG family and can be activated by hypoxia.^222,223^ The AP-1 (activator protein-1) complex is formed from heterodimers of either the Jun or Fos proteins or homodimers of Jun proteins.^223,224^ The AP-1 binding sequence is a common component of transcriptional regulatory elements that can drive the activation of multiple target genes during hypoxia, including tyrosine hydroxylase (TH), which encodes an important enzyme in catecholamine synthesis.^225,226^ Because TH is the rate-limiting enzyme for catecholamine synthesis, it is possible that IH-induced TH activation partially induces an increase in catecholamine levels in the body,^227,228^ leading to a chronic increase in sympathetic activity.^229^ In addition, the upregulation of AP-1 is involved in the expression of adhesion molecules and inflammatory cytokines, suggesting that AP-1 is also involved in OSAS-induced systemic chronic inflammation.^230,231^ Yuan et al. reported that IH increased c-fos mRNA expression in PC12 cells in a stimulation-dependent manner, and the IH-induced increase in c-fos mRNA was due in part to an increase in c-fos transcriptional activation.^152^ Further experiments showed that point mutations in the c-fos promoter indicated that the serum-responsive element and Ca^2+^ response element are vital for IH-induced c-fos promoter activation.^152^ Interestingly, several studies have found that IH increases the expression of c-fos mRNA in PC12 cells. However, continuous hypoxia exposure (equal to the accumulated time of IH) had no effect.^152,232^ In addition, prolonged continuous hypoxia was able to activate c-fos mRNA, and when the c-fos gene was activated by continuous hypoxia, the expression level of c-fos mRNA returned to the control level within 30 min after termination of hypoxic stimulation. Interestingly, c-fos mRNA levels remained high 5 h after the end of IH.^233^ Another study found that c-fos mRNA continued to increase for at least 3 h after IH intervention but returned to normal levels within 1 h after continuous hypoxia cessation,^152^ suggesting that different hypoxia modes have significant differences in the regulation of c-fos mRNA. Long-lasting activation of c-fos mRNA by IH is closely related to IH-induced carotid body sensory activity^234^ and respiration.^235,236^ A major difference between IH and continuous hypoxia is that IH has a reoxygenation phase, which is absent during continuous hypoxia. Therefore, it has been proposed that the generation of ROS by IH during the reoxygenation phase may mediate the regulation of c-fos mRNA. The amount of c-fos mRNA expression activated by IH was reported to be dependent on the duration of reoxygenation after hypoxia but not on the duration of hypoxia.^152^ Superoxide ion scavengers [manganese tetrakis methyl porphyrin pentachloride (MnTMPyP)] could inhibit the upregulation of c-fos mRNA and attenuate the transcriptional activation of AP-1 induced by IH.^152,237^ Studies have shown that the Ca^2+^ signaling pathway is involved in the hypoxic activation of the c-fos gene and AP-1 in PC12 cells.^193,222^ RT‒PCR and reporter gene assays showed that hypoxia enhanced c-fos mRNA and promoter activity, which were inhibited by the Ca^2+^ chelator BAPTA-AM or L-type Ca^2+^-channel blocker, while the L-type Ca^2+^-channel agonist BAYK8644 enhanced c-fos gene activation by hypoxia.^193^ Further immunoblot analysis showed that hypoxia increased the expression of CaMKII protein in PC12 cells, whereas the CaMKII inhibitor inhibited hypoxia-induced stimulation of the c-fos promoter.^193^ Ectopic expression of CaMKII mutants was also able to stimulate c-fos promoter activity under normoxic conditions. In addition, hypoxia-induced phosphorylation of CREB at the serine residue,^133^ and CaMKII inhibitors inhibited this effect.^193^ In summary, Ca^2+^-dependent signaling pathways play a vital role in hypoxia-regulated c-fos gene expression (Fig. 3 ⑥).

### Mechanisms associated with altered carotid body function in response to IH

Patients with IH due to recurrent apnea, as well as IH-exposed rodents, develop autonomic abnormalities, including enhanced hypoxic ventilatory responses, elevated plasma catecholamines, persistent activation of the sympathetic nervous system, and systemic hypertension.^238,239^ The acute response to hypoxia, which occurs within seconds to minutes, is entirely dependent on the oxygen-sensitive capacity of peripheral arterial chemoreceptors, particularly the carotid bodies.^240–242^ Studies have shown that carotid body chemoreceptor are the “front line” defense system to detect alterations in arterial blood oxygen during apnea, which is more sensitive and rapid than other respiratory chemoreceptors, such as central chemoreceptors.^243–245^ This is because the time for oxygen to diffuse from the lung to the carotid body (6 s) is shorter than the time to reach the central region, and thus, the carotid body has already responded to hypoxia before the hypoxic stimulus is felt in the central region. Given its location and functional properties, IH-induced carotid body activation is closely related to autonomic dysfunction.

When it is starved of oxygen, the body actively begins to increase ventilation within a few minutes. This physiological response to increase ventilation due to oxygen deficiency is called the hypoxic ventilatory response (HVR).^246^ OSAS patients and IH-exposed rodents exhibit enhanced HVR,^247,248^ a hallmark of the carotid body chemoreflex.^249,250^ In a rodent model, awake rats were exposed to IH (5% O_2_ for 15 s, 21% O_2_ for 5 min; 9 sessions per hour, 8 h per day for 10 days). Efferent phrenic nerve activity was used as an indicator of neural respiration to assess HVR. The results showed a 38% increase in baseline minute neural respiration and a 56% increase in ventilatory stimulation induced by acute hypoxia (12% inspired O_2_ fraction).^233^ As reported in another experiment, there was no significant increase in HVR in rats exposed to 30 days of IH. It is possible that HVR becomes adaptive after 30 days compared to 2 weeks of IH.^251^ Exposure of experimental animals (cats,^252^ dogs,^253^ rats,^254^ and goats^255^) and humans^256,257^ to repeated hypoxia promotes a compensatory and sustained (>1 h) increase in respiratory motor activity. This prolonged respiratory activation in response to IH is often referred to as respiratory long-term facilitation (LTF),^258,259^ which is considered to be a marker of IH because a similar duration of continuous hypoxia does not result in prolonged respiratory activation. It was found that rats exposed to IH for 10 days showed a significant enhancement in LTF of respiratory motor output.^233^ It has been hypothesized that LTF prevents collapse by increasing the tone of the upper airway and that enhanced LTF may contribute to increased basal ventilation in patients with OSAS as well as in animals exposed to IH. Afferent input to the carotid body may be critical for LTF in respiratory motor output resulting from IH. Therefore, a group of researchers further investigated the effect of IH on chemoreceptor sensory discharge in the carotid body of rats, and anesthetized rats were subjected to 10 sessions of hypoxia (12% O_2_ for 15 s) followed by 5 min of reoxygenation.^260^ Interestingly, when this hypoxic pattern was repeated in animals subjected to IH for 10 days, it resulted in a prolonged elevation of baseline carotid somatosensory activity for nearly 1 h.^260^ These observations suggest that IH induces novel functional plasticity of the carotid body, leading to LTF in sensory discharge. However, sensory LTF plays an important role in reflex activation of the sympathetic nervous system and sustained daytime hypertension,^261,262^ and ablation of the carotid body reduces sympathetic activation and hypertension in intermittently hypoxic rats.^263,264^

ROS, which are produced during the reoxygenation phase of IH, may play a vital role in eliciting changes in carotid body activity induced by IH.^265,266^ In contrast to rats exposed to IH, the response of the carotid body was found to be blunted under continuous hypoxia; additionally, there was no induction of LTF in the sensory discharge of the carotid body under continuous hypoxia.^260^ Physiological studies showed that antioxidants (MnTMPyP and N-acetylcysteine) could ameliorate IH-induced plasma catecholamine elevation^227^ and decrease hypoxia sensitivity in the carotid body, and the magnitude of the LTF during sensory discharge was also significantly attenuated.^204,249^ Several studies have also confirmed that intervention with ROS scavengers during exposure of rats to IH could normalize carotid body activity and improve IH-induced hypertension.^227,234,267^ Increased sensitivity of carotid body chemoreceptors to hypoxic chemotherapy may involve endothelin (ET) and ET receptors,^268–270^ which are expressed in glomus cells (oxygen-sensitive type I cells) and blood vessels in the carotid body.^271^ ET acts on two receptors, the ETA receptor and the ETB receptor.^272^ In rodents exposed to IH, quantitative RT‒PCR confirmed a gradual increase in ET and ETA expression in type I cells and a time-dependent increase in hypoxia-induced carotid receptor activity. The application of a specific ETA antagonist could inhibit or attenuate hypoxia-induced carotid sensory discharge.^272^ In cats exposed to chronic IH for 4 days, ET-1 expression increased approximately 10-fold in the carotid body, while plasma ET-1 levels were unchanged, and the ETA/ETB receptor antagonist inhibited the chronic IH-induced increase in the carotid body hypoxic chemosensory responses.^270^ Another study found that the administration of MnTMPyP prevented the IH-induced elevation of ROS, basal release of ET-1 levels, and ETA receptor mRNA and augmented sensory responses. These observations suggest that the IH-induced increase in sensory responses involves a ROS-mediated increase in ET-1 release and upregulation of ETA receptor mRNA.^273^ A recent study explored chronic IH to increase carotid body chemosensory sensitivity via the ET-1 receptor signaling pathway.^274^ PKC, PLC, or p38 MAPK antagonists were used to elucidate the signaling pathways involved. The results showed that after chronic IH exposure, the protein levels of p38 MAPK and PKC were increased, and the expression of ETA and ETB receptors was upregulated in the carotid body, but only ETA was involved in ET-1-induced carotid body chemosensory sensitivity.^274^ It was confirmed that ETA receptor-mediated PLC, PKC and p38 MAPK signaling pathways were responsible for chronic IH-induced carotid body chemosensory sensitivity, and Ca^2+^ influx was also involved in the increase in carotid sinus nerve activity.^274^ In addition to ET-1, the renin-angiotensin system is also strongly associated with enhanced carotid body chemosensory sensitivity. Angiotensinogen mRNA and protein have been found to be present in type I cells. Similar to ET-1, IH increased the transcriptional and posttranscriptional expression of angiotensin II type 1 receptor (AT1) in the carotid body.^275^ Interestingly, the study by Lam and Leung et al.^276^ found that angiotensin II was able to act directly and enhance carotid body chemosensory sensitivity, rather than being mediated by altered arterial pressure or blood flow, and angiotensin II enhances carotid sinus nerve activity in the carotid artery in vitro. Based on the current study, we hypothesize that IH induces the production of sensory LTF in the carotid body through ROS/Ca^2+^/AT signaling to increase the sensitivity of the carotid body to hypoxic chemotherapy, which may be an important molecular mechanism of sympathetic activation after IH (Fig. 3 ⑦).

Type I cells in carotid bodies are derived from neurons and are the primary oxygen-sensing cells. Available evidence indicates that type I cells are the initial site of sensory transduction and that they release an excitatory neurotransmitter in response to hypoxia, acting on nearby afferent nerve endings and thus resulting in increased sensory discharge.^240,277^ One hypothesis suggests that heme and/or redox-sensitive enzymes are oxygen sensors and that biochemical events associated with heme proteins trigger transduction cascades,^278^ which leads to increased cytosolic Ca^2+^ concentrations and evokes neurotransmitter release in type I cells. An alternative hypothesis suggests that K+ channel proteins are oxygen sensors and that inhibition and subsequent depolarization of this channel is the initiating event in transduction.^278,279^ ROS may enhance the hypoxia-induced increase in intracellular Ca^2+^ concentration in type I cells by affecting voltage-gated Ca^2+^ channels, thereby enhancing sensitivity to hypoxia. One study showed that ROS enhanced the increase in intracellular Ca^2+^ concentration in PC12 cells in response to depolarizing stimulation, but the specific triggering mechanism is unclear.^280^

Recent studies have shown that the sensing of hypoxia in the carotid body requires an O_2_-dependent interaction between hydrogen sulfide (H2S) and carbon monoxide (CO).^281–285^ CO produced by heme oxygenase-2 (HO-2) in the carotid body induces a signaling pathway.^286^ CO inhibits the CSE (cystathionine γ-lyase) activity of the carotid body through protein kinase G (PKG)-dependent phosphorylation of serine residue 377, thereby inhibiting hydrogen sulfide (H2S) synthesis and leading to the inhibition of carotid body activity.^283^ Interestingly, the IH-increased H2S production was due to ROS-dependent inactivation of HO-2 that reduced CO production in the carotid artery, which in turn reduced the inhibitory effect of PKG on CSE phosphorylation,^283^ thereby increasing the H2S concentration and stimulating its neural activity.^287^ Rodents exposed to IH showed a significant increase in the H2S concentration in the carotid body, and this effect was abolished in rats treated with the CSE inhibitor L-propargylglycine (L-PAG).^287^ Furthermore, CSE-deficient mice showed a significant reduction in basal H2S levels in the carotid body,^281^ suggesting that IH increased CSE-dependent H2S production. HO-2 knockout mice exhibit more abundant CSE-derived H2S in carotid bodies and enhanced carotid body chemosensitivity, and CSE inhibitors prevent OSAS in HO-2 knockout mice.^288^ The carotid body of IH-exposed rats showed reduced CO levels, PKG activity, and CSE phosphorylation, whereas all of these effects were abolished after administration of the membrane-permeable ROS scavenger MnTMPyP.^287^ Therefore, we hypothesized that the activation of H2S signaling in the carotid body under IH is also a key trigger of sympathetic activation and hypertension (Fig. 3 ⑧). In addition, increased H2S may mediate ROS-induced intracellular Ca^2+^ elevation (Fig. 3 ⑨). Previous studies have shown that voltage-gated Ca^2+^ channels (VGCCs) are essential for hypoxia-induced Ca^2+^ elevation in type I cells,^289,290^ with L-type (high-voltage-activated channel) VGCCs mediating the majority of the hypoxia-induced Ca^2+^ influx.^291,292^ A recent study detailed the role of T-type (low-voltage-activated channel) VGCCs in the carotid body and found that the mRNA encoding the α1H subunit and α1H-protein is highly expressed in rat carotid body type I cells, implying that CA_V_3.2 is the major T-type VGCC isoform in the carotid body.^293^ Mibefradil and TTA-A2, as selective blockers of T-type VGCCs, significantly reduced the hypoxia-induced increases in intracellular Ca^2+^ concentration, catecholamine secretion from type I cells, and sensory excitation of the carotid body.^293^ Studies have also confirmed that H2S, dependent on CSE production, is required for VGCC-mediated Ca^2+^ influx in type I cells^294^ and carotid body sensory nerve excitation.^281,284^ Interestingly, similar to hypoxia, the H2S donor NaHS increased the intracellular Ca^2+^ concentration and carotid body nerve activity, while these effects were significantly attenuated in CA_V_3.2 knockout mice.^293^ In wild-type mice, TTA-A2 significantly reduced the response of type I cells and carotid body sensory nerves to hypoxia, and these effects were abolished in CSE knockout mice.^293^ Based on the present findings, we hypothesized that the highly expressed CA_V_3.2 T-type VGCCs in type I cells are involved in H2S-mediated Ca^2+^ influx and Ca^2+^ secretion, as well as the response of the carotid body to hypoxia. However, whether other types of calcium channels also play these roles in IH and hypoxia is unknown, and the types of oxygen-sensitive channels need to be further explored in the future.

### Mechanisms of OSAS-induced gut dysbiosis

In normal physiological states, there is a mutually beneficial relationship between the host and the gut microbiota. The host provides nutrients and a living environment for the microbiota, while bacteria help maintain the host immune response, act as a barrier against invading pathogens, and provide nutrients to the host.^295,296^ This balanced relationship may be disrupted by changes in the composition of the microbiota, known as dysbiosis. Current studies have found that gut dysbiosis might play a role in OSAS-associated morbidities, such as systemic hypertension,^297–300^ metabolic disorders,^301–303^ neurological diseases,^304^ COVID-19,^305^ and atherosclerotic heart disease.^306^ The gut is the largest immune organ and the largest microecosystem in the human body. The gut microbiota contains at least 1500 species of microorganisms with more than 100 trillion bacteria,^307,308^ and 70% of lymphoid tissue is present in the gut and forms gut-associated lymphoid tissue.^309^ The five most common bacterial phyla inhabiting the colon are *Actinomycetes*, *Bacteroides*, *Proteus*, *Firmicutes*, and *Cerrucomicrobia*.^310^ Bacteroides and Firmicutes account for 90% of the bacteria in the colon.^311^ The beneficial and healthy Bacteroidetes (gram-negative) include *Lactobacillaceae*, *Ruminococcaceae*, *Erysipelotrichaceae*, *Bifidobacteriaceae*, and *Clostridium*, which play key roles in carbohydrate and fiber fermentation. This process produces short-chain fatty acids [SCFAs (butyrate, acetate, and propionate)], which provide the main source of nutrition and energy for colonic cells and regulate the immune system.^312–314^ On the other hand, *Desulfovibrio*, *Prevotella*, *Lachnospiraceae*, and *Paraprevotella* species, which belong to *Firmicutes*, have local (gut) and systemic harmful characteristics and are capable of disrupting the structural integrity of the gut barrier.^315,316^ Interestingly, an increased *Firmicutes*/*Bacteroidetes* (F/B) ratio has been shown to be a hallmark of gut dysbiosis in almost all animal studies using similar IH exposure models.^310,316,317^

It is well known that the core of the gut contents is hypoxic, but studies have shown that there is a gradient in the oxygen concentration of the microbiota in the range of ≈150–200 μm near the gut epithelium^318^ and that the oxygen concentration has an effect on the microbiota.^319^ In a mouse model of IH intervention, it was found that IH induced a periodic hypoxia/reoxygenation pattern in arterial blood and the lumen of the small intestine. It is possible that there is a physiological process involving oxygen diffusion from the epithelial capillaries into the gut lumen, and a periodic pattern of hypoxia/reoxygenation could be observed within 200 μm of the intestinal epithelial barrier^316^; that is, IH translates into a hypoxia/reoxygenation pattern in the proximal intestinal epithelial feces (<200 μm). Under these conditions, we hypothesized that an increased duration of hypoxia would favor the survival of obligate anaerobes and that the biological diversity of the gut microorganisms might be altered. In fact, some studies have also confirmed that IH exposure causes changes in the relative abundance of aerobic bacteria in mice that mimic moderate OSAS and causes an increase in the abundance of obligate and facultative anaerobes.^319^ In addition, dysbiosis was characterized by a changed F/B ratio in many experiments.^320,321^ Given that arousal is an important component in the pathogenesis of OSAS, a recent study showed that when mice were exposed to sleep fragmentation, it resulted in significant changes in the microbiota, including an increase in *Firmicutes* and a decrease in *Bacteroidetes* compared with those of control mice.^322^ Another consequence of arousal is increased sympathetic activity and catecholamine release,^323^ and catecholamines could significantly increase the growth of certain bacterial species.^324,325^ Adrenergic stimulation of enteric neurons regulates intestinal motility and ion transport, thereby altering the microbiota.^326,327^ In addition, adrenergic release from the intestinal epithelial layer disrupts the integrity of the epithelial barrier.^327^

**Fig. 4:**
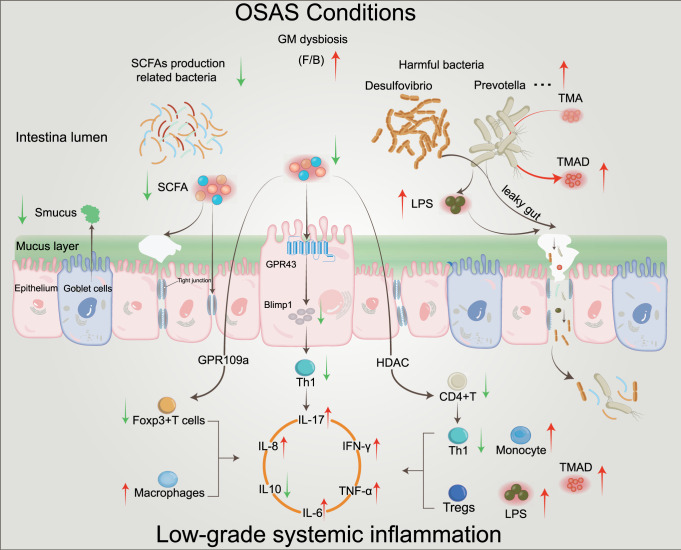
OSAS-induced low-grade systemic inflammation by mediating gut dysbiosis. The increased F/B ratio is a hallmark of gut microbiota dysbiosis, which is mainly characterized by a decrease in SCFA production-related bacteria and an increase in harmful bacteria. Decreased mucus secretion and mucin synthesis by dermal goblet cells disrupt the integrity of the intestinal barrier. The intestinal epithelium is dysfunctional due to inadequate nutrition, manifesting as reduced mucus production, decreased mucin secretion, and disrupted intestinal barrier integrity. Increased abundances of *Prevotella* and *Desulfovibr*io produce lipopolysaccharide and promote the degradation of mucin, increasing intestinal permeability and leading to a “leaky gut”, which triggers an intrinsic and adaptive immune response that induces low-grade inflammation in the body. *Prevotella* converts nutrients containing TMA into TMAO, which promotes inflammation. The reduced ability of SCFAs to activate GPR43, GPR109a, and HDAC results in diminished anti-inflammatory and increased proinflammatory capacity. GM gut microbiota, F/B Firmicutes/Bacteroidetes, SCFAs short-chain fatty acids, TMA trimethylamine, TMAO trimethylamine oxide, LPS lipopolysaccharide, HDAC histone deacetylase, GPR G-protein-coupled receptor, Blimp-1 maturation protein-1

In OSAS patients, IH leads to ischemia-reperfusion injury of the intestinal mucosa and insufficient oxygen supply to the intestinal mucosa, resulting in changes in the structure and abundance of the gut bacteria and destruction of the integrity of the intestinal barrier.^328–330^ *Prevotella* and *Desulfovibrio* belong to the specific bacterial phylum *Firmicutes*, and the abundances of both bacteria increased significantly with IH exposure,^316,322^ exhibiting mucin-degrading features. The sulfate released during mucin degradation by *Prevotella* is cleared by *Desulfovibrio*, a process that further promotes mucin degradation and increases gut permeability.^316,331^ Disruption of the intestinal wall membrane integrity produces a small-molecule protein (plasma intestinal fatty acid-binding protein) that is considered to be a highly sensitive marker of the ischemic intestinal mucosa.^332–334^ Interestingly, plasma intestinal fatty acid-binding protein was found to be significantly elevated in OSAS patients.^332,335^ In addition, it has been found that the plasma D-lactic acid level is closely related to the permeability and degree of damage of the intestinal mucosa in patients with OSAS and is positively correlated with AHI.^336^ Dysbiosis of the gut microbiota reduces the levels of butyrate and acetate, causing intestinal mucosal nutritional disorders, which could lead to a dysfunctional epithelium.^312,313,337^ In addition, repeated hypoxia/reoxygenation cycles also damage the epithelium.^338,339^ Eventually, the tight junctions between colonic epithelial cells are destroyed, resulting in a “leaky gut.” As *Prevotella* produces endotoxin (lipopolysaccharide)^340^ and other bacterial components that leak from the gut into the blood circulation, it stimulates the release of inflammatory mediators,^341^ such as interleukin (IL)-6 and tumor necrosis factor (TNF)-α, through monocyte recruitment and Toll-like receptor activation,^342^ thereby aggravating systemic inflammation.^322,343^ Interestingly, a positive correlation was found between the abundance of the mucin-degrading bacterium *Desulfovibrio* and plasma lipopolysaccharide in IH-exposed mice.^344^ In addition, *Prevotella* converts nutrients (choline and l-carnitine) containing trimethylamine (TMA) into trimethylamine oxide (TMAO), which promotes inflammation, thrombosis, and the uptake of LDL by macrophages^345^ and contributes to hypertension^346,347^ and atherosclerosis.^348–351^ Multiple gut microfloral analyses demonstrated a reduction in bacteria associated with SCFA production in OSAS animal models^320,321^ and OSAS patients.^328^ SCFAs play an important role in maintaining intestinal integrity. Butyrate is a major source of energy and nutrition for enterocytes.^352^ An in vitro study has shown that butyrate enhances the expression of tight junction proteins, which are located transversally between epithelial cells,^353^ thereby increasing transepithelial resistance, maintaining gut integrity, and preventing gut permeability.^354^ Butyrate and propionate could induce the secretion of some mucin glycoproteins necessary for the construction of a mucus layer (which separates the colonocytes from the lumen) to protect intestinal epithelial cells.^355^ In addition, acetate enhances the differentiation of intestinal epithelial goblet cells and the secretion of mucus,^356^ which is beneficial for increasing the tight junction of enterocytes and improving the immune defense ability of enterocytes^357^ to inhibit lipopolysaccharide and bacteria from the gut entering into the systemic circulation. As hormone signaling molecules, SCFAs regulate immunity directly or indirectly through host metabolism through specific receptors.^358,359^ Butyrate can act on signal transducers of Th1 cells (T-helper 1 cells) and mTOR, an activator of transcription, and can upregulate B lymphocyte-induced maturation protein-1 (Blimp-1). Butyrate may induce the production of highly differentiated Th1 cells by acting on G-protein-coupled receptor 43 (GPR43) on intestinal epithelial cells to cause them to then secrete IL-10 and inhibit the excessive inflammatory response of Th cells.^360^ Butyrate also activates GPR109A, induces Treg and T-cell differentiation to produce IL-10 and inhibits intestinal inflammation by enhancing the anti-inflammatory properties of colonic macrophages and dendritic cells (DCs).^361,362^ The normal gut microbiota and its metabolites contribute to the regulation of Th17/Treg cell balance. Studies have found that SCFAs promote the proliferation and differentiation of Treg cells via epigenetic mechanisms.^363,364^ It has been confirmed that Th17/Treg cell imbalance is associated with the development of several disorders, and it is interesting to note that OSAS patients exhibit an increase in the number of Th17 cells^365^ and a significantly increased Th17/Treg cell ratio.^366^ Further studies showed that butyrate treatment of naive T cells could enhance histone H3 acetylation levels in the promoter and noncoding regions of the Foxp3 (forkhead Box p3) gene,^363^ induce naive CD4^+^ T cells to differentiate into peripheral Tregs, which secrete IL-10, and suppress the excessive immune response induced by Th1 and Th17 cells.^360,367–369^ Propionate and butyrate can downregulate the histone deacetylase (HDAC) activity of T cells to regulate immune function.^370^ This regulation might increase the phosphorylation of ribosomal protein S6, a target of the mTOR pathway, and induce the acetylation of p70 S6 kinase (S6K) and further phosphorylation of S6,^371^ ultimately promoting the differentiation of CD4^+^ T cells and the secretion of IL-10, IFN-γ, and IL-17.^372^ Interestingly, SCFAs can cross the blood‒brain barrier through the circulatory system, affect the growth and development of microglia, control their function and maturation, and enhance immunity and immune defense of the brain.^373,374^ There is increasing evidence that butyrate may provide neuroprotection by reducing microglial activation, which in turn decreases the levels of proinflammatory mediators and increases the levels of anti-inflammatory mediators.^375^ SCFA treatment also ameliorated the defective morphology and maturation of microglia in germ-free animals.^373^ Apparently, SCFAs have an immunomodulatory capacity not only in the gut and periphery but also in the nervous system. The mechanisms of OSAS-induced gut dysbiosis are shown in Fig. 4.

### IH-induced oxidative stress in OSAS

**Fig. 5:**
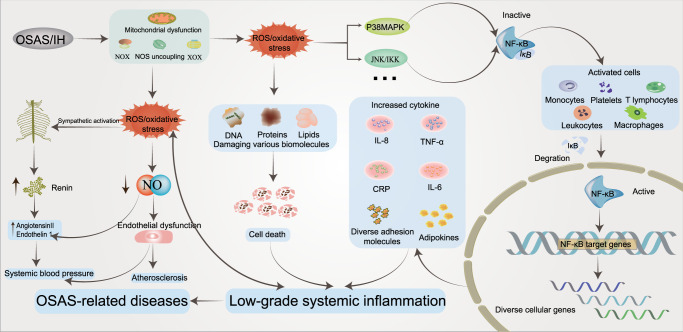
Schematic demonstrating the central role played by oxidative stress and inflammation in OSAS. OSAS/IH induces ROS production by inducing mitochondrial dysfunction, activating NOX and XOX, and inducing NOS uncoupling, which results in oxidative stress. The interaction between ROS and NO further promotes oxidative stress and diminishes the bioavailability of NO, thus promoting endothelial dysfunction and inflammation, which is closely related to hypertension, atherosclerosis, and hypercoagulability. Increased ROS-dependent sympathetic activation enhances renin levels, which leads to an increase in angiotensin II, endothelin 1, and hypertension. As a second messenger, ROS can activate multiple signaling pathways (MAPK, JNK), which in turn activate NF-κB and then induce the activation of nuclear transcription factors in a variety of cells. As the main switch of the inflammatory response, NF-κB plays an important role in the pathological process of OSAS, activating and entering the nucleus, regulating the transcription of many kinds of cells (immune cells), causing an increase in cytokines and participating in the inflammatory process of cells. In addition, elevated ROS can damage intracellular macromolecular substances (DNA) and cause cell death. Various pathological processes coordinate with each other and induce low-grade inflammation in the body, which is closely related to the occurrence and progression of a variety of diseases. ROS reactive oxygen species, NOX NADPH oxidase, NOS uncoupling nitric oxide synthase uncoupling, XOX xanthine oxidase, NOS nitric oxide synthase, JNK c‐Jun N‐terminal kinase, MAPK mitogen-activated protein kinase, NF-κB nuclear factor kappa B

In recent years, increasing evidence has implicated oxidative stress as a fundamental component of OSAS pathophysiology, which is manifested by increased ROS production and decreased antioxidant capacity.^2,376^ Oxidative stress is defined as a break in the balance between oxidant-generating systems and antioxidant defense mechanisms, and the oxidative stress associated with OSAS is due to the production of ROS exceeding the antioxidant supply.^376^ Repeated breathing cessation is characteristic of OSAS, a severe hypoxic episode followed intermittently by rapid blood oxygenations that could be considered to be similar to repeated ischemia-reperfusion events, which affects cellular components and functions, resulting in increased ROS production. In the reperfusion period, the flux of excess ROS can alter their biological functions and induce various pathologies by damaging various biomolecules, such as proteins, lipids, carbohydrates, and DNA.^1,2,377,378^ In OSAS, the main sources of ROS for these pathologies are derived from damaged mitochondria, activated inflammatory cells, or superoxide production by activated enzyme systems, such as xanthine oxidase, nitric oxide synthase uncoupling and NADPH oxidase^2^ (Fig. 5). Hypoxia and reoxygenation might also induce complex metabolic and molecular changes, which include changes in gene expression and changes in energy metabolism.^230^ The disruption of oxidant-producing systems and antioxidant defense mechanisms may also result from decreased antioxidant capacity. A decrease in antioxidant capacity resulting in an increased oxidative stress load has also been described in OSAS. For example, the total antioxidant capacity of serum is decreased in OSAS patients.^379^

Oxidative stress initiates a vicious cycle that facilitates the increased production of inflammatory cytokines, producing a systemic inflammatory state that increases vascular cell adhesion molecules and promotes sympathetic activation and vagal activation.^1,379^ Sympathetic activation stimulates the renin-angiotensin-aldosterone system (RAAS), which leads to increased levels of angiotensin II and aldosterone in the blood (Fig. 5). In addition, increased sympathetic tone is the key mediator of disrupted glycemic and insulin homeostasis, which may contribute to the development of metabolic risk factors in OSAS.^2,380^ Studies have found excessive ROS and increased expression of adhesion molecules and inflammatory cytokines, which reduce nitric oxide (NO) activity.^2^ The main consequences are endothelial dysfunction and hypercoagulability, which are identified as pathogenic mechanisms involved in different clinical and experimental models and affect various conditions and diseases (Fig. 5). However, in each disease, the results may differ according to the most affected organ or cellular function.^379^ It is estimated that more than 100 pathologies are associated with ROS and oxidative stress. Among them are cerebrovascular disease, cardiovascular disease, metabolic syndrome, type 2 diabetes, carcinogenesis and metastasis, inflammatory diseases (such as glomerulonephritis), atherosclerosis, and hypertension.^2^

A large body of evidence indicates that under normal physiological conditions, ROS function as signaling molecules, consistently described as regulators of signal transduction and as second messengers in many signaling pathways in all cells.^381^ Evidence regarding the capacity of ROS as signaling molecules is increasing. ROS regulates biological processes such as proinflammatory, profibrotic, cell proliferation, differentiation, migration, and apoptosis without triggering a requirement for macromolecular damage.^382,383^ Disruption of the ROS balance may activate a plethora of signaling pathways and inhibit others, affecting gene expression and protein function and leading to changes in signaling output, enzymatic activity, membranes, and intercellular communication.^383–385^ We present here a few examples of signaling targets.

Increased intracellular ROS were implicated in the PI3K cascade, c‐Jun N‐terminal kinase (JNK), and MAPK pathways that might induce the activation of multiple nuclear transcription factors (Fig. 5), such as nuclear factor kappa B (NF-κB), AP-1, redox factor-1 (Ref-1), HIF-1α, sterol regulatory element binding proteins (SREBPs), p53 and GATA-4.^383,386^ NF-κB, as a master switch in inflammation, is of special interest in the pathological process of OSAS, which is subject to complex regulation involving many regulatory molecules. At the same time, it orchestrates the production of adhesion molecules, inflammatory cytokines, and adipokines in OSAS.^387,388^ In addition, AP-1 expression was upregulated in cultured PC12 cells exposed to IH. Given that the upregulation of AP-1 is similar to that of NF-κB, AP-1 might also be involved in the pathogenesis of OSAS.^152,230^ However, the pathways of activation are not yet fully elucidated. HIF-1α is a transcription factor that plays a major regulatory role in the transcriptional response to decreased oxygen levels, which is essential for oxygen homeostasis and the adaptive response to hypoxia,^381,389^ and has been found mainly in several experimental models of IH in tissue culture as well as in rodents exposed to chronic IH.^390^ In addition, it has been stated above that the transduction signals that activate HIF-1α under IH conditions are distinct from those activated by sustained hypoxia.^381^ IH may cause worse HIF-1α stability, resulting in the activation of NF-κB-induced inflammation, possibly as a result of oxidative stress.^391^ In addition, it is becoming increasingly clear that there is a large degree of crosstalk between HIF-1α and the NF-κB pathway, and recent studies suggest that the NF-κB pathway plays a key role in inflammation induced by sustained hypoxia.^392^ OSAS has been shown to activate redox signaling, which may contribute to several systemic and cellular functional changes (including changes in blood pressure, increased release of neurotransmitters, and alterations in sleep and cognitive function) that are associated with the activation of second messenger pathways and HIF-1α, which is potentially important in OSAS pathology.^148,393^ SREBPs are a group of transcription factors affected by redox imbalance and oxidative stress that regulate the expression of genes required to maintain lipid homeostasis.^394^ In an experimental model of IH, the SREBPs activating genes regulating lipid metabolism were shown to be upregulated.^395,396^ Recently, a series of elegant studies has shown that lipid peroxidation and atherosclerosis are closely associated with the severity of chronic IH, and SREBP pathway-mediated hyperlipidemia was observed in this model.^397,398^ Additional transcription factors that are redox-sensitive and could possibly be implicated in OSAS pathology include NRF2-Keap1, which regulates antioxidant genes with a role in maintaining redox homeostasis.^399^

### IH-induced systemic inflammation in OSAS

IH appears to be an important mechanism triggering inflammatory pathways.^400^ As outlined above, the main mechanisms of OSAS are hypoxia and oxidative stress, which are potent inducers of a cascade of inflammatory pathways. Furthermore, several studies have confirmed that inflammation also plays a crucial role in the occurrence and development of OSAS^401^ (Fig. 5). IH is hypothesized to activate the NF-κB-mediated inflammatory pathway that induces the overexpression of adhesion molecules [such as E- and P-selectin, intracellular adhesion molecule (ICAM) and vascular cell adhesion molecule (VCAM)], adipokines and proinflammatory cytokines [TNF-α, IL-1, IL-6, IL-8, and C-reactive protein (CRP)].^386,402^ Activation of these inflammatory pathways promotes the activation of endothelial cells, immune cells (circulating leukocytes, monocytes, and T lymphocytes), and platelets.^403^ These activated cells can further promote oxidative stress and injury by releasing ROS and increasing the expression of adhesion molecules on leukocytes, platelets, and endothelial cells, thereby exaggerating the inflammatory response^386,404^ (Fig. 5). In OSAS pathophysiology, as well as in the conditions and comorbidities that aggregate with it, the presence of inflammation can be considered a potential contributor to OSAS.^405^

Cytokines are intracellular and extracellular soluble mediators that, by interacting with various transcription factors in a very complex and intermingled network, regulate both the innate and acquired immune systems, orchestrating immune cells and inflammatory responses.^406^ They stimulate cells to secrete inflammatory cytokines, activate and recruit macrophages, promote the proliferation of smooth muscle cells, interfere with nitric oxide production, and activate endothelial cells to cause vascular dysfunction.^403^ TNF-α synthesized by macrophages is a cell signaling proinflammatory cytokine that is involved in host defense, immune mechanisms, and the pathogenesis of different infections and participates in a large number of signaling events that, in turn, lead to necrosis and apoptosis.^407^ In patients with OSAS, circulating TNF-α levels are not only elevated in plasma or serum^408^ but are also elevated in monocytes and various cytotoxic T lymphocytes.^379^ In addition, TNF-α stimulates NF-κB activity, promoting increased expression of VCAM in endothelial cells,^409^ which enables enhanced monocyte adhesion to the endothelium, triggers inflammatory responses in endothelial cells, and promotes the initiation and progression of atherosclerosis. Interestingly, activation of inflammatory pathways via upregulation of NF-κB has recently been found in monocytes from patients with OSAS^410,411^ (Fig. 5). Several studies have highlighted the persistence of a state of systemic chronic low-grade inflammation in patients with OSAS, mainly characterized by increased levels of TNF-α, IL-6, IL-8, and CRP.^407,408^ The major proinflammatory cytokines (TNF-α, IL-6, and IL-8) that activate NF-κB and AP-1 are regulated by oxygen tension and free radicals.^412^ Conversely, these cytokines can further activate inflammatory transcription factors and enhance inflammatory responses by activating various blood cells and endothelial cells. Adhesion molecules are cell surface proteins that play a key role in intercellular associations and are considered to be a major part of the inflammatory response against hypoxia. When facing various stimuli, such as hypoxia/reoxygenation and OSAS, adhesion molecules, and cytokines are upregulated in blood leukocytes and endothelial cells, which promote endothelial cell injury.^384^ CRP not only upregulates the transcriptional activity of NF-κB but also promotes the expression of ICAM and VCAM, which induces monocyte-endothelial cell adhesion.^413^ Thus, it is clear that CRP is not only an inflammatory marker but also a functional regulator that might contribute to the development of inflammation in OSAS through oxidative stress.

### Other IH-induced signaling pathways in OSAS

**Fig. 6:**
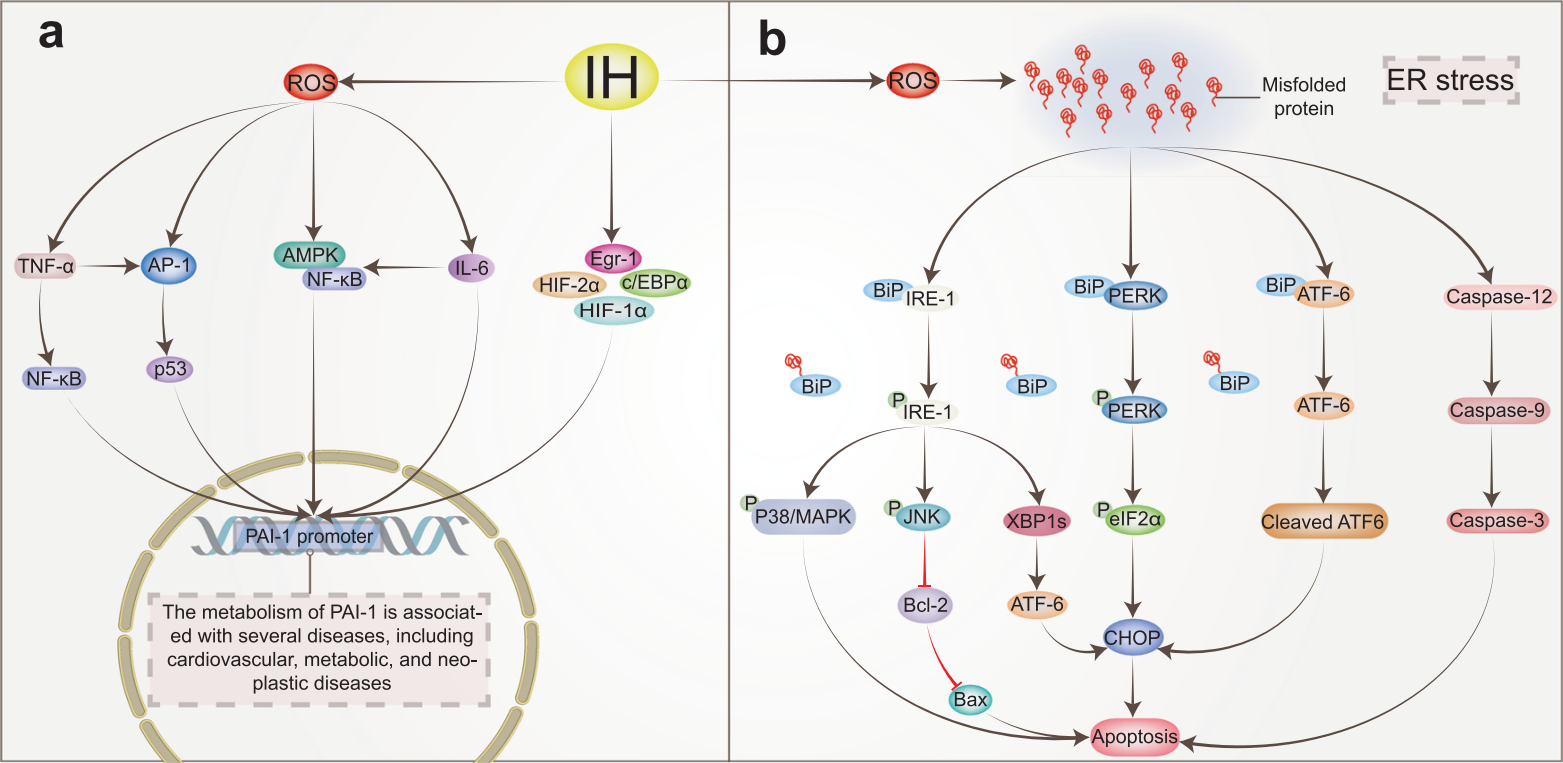
Other IH-induced signaling pathways in OSAS. IH regulates PAI-1 transcription through multiple pathways (**a**). IH could induce ROS, which in turn activated TNF-α, AP-1, AMPK/NF-κB pathway, and IL-6. In addition, IH could also promote the expression of Egr-1, HIF-2α, HIF-1α, and C/ΕBPα, ultimately upregulating the transcription of PAI-1. Upregulation of PAI-1 is associated with the development of IH-related disorders. Possible mechanisms of apoptosis induced by IH (**b**). IH could induce the generation of ROS, which in turn causes ER stress manifested by the production of misfolded proteins that bind BiP released from IRE1, PERK, and ATF6. After BiP release, IRE1, PERK, and ATF6 are activated. The activated IRE1, PERK, and ATF6 further activate their respective downstream pathways to ultimately upregulate the expression of CHOP and promote cell apoptosis. In addition, ER stress activates caspase-12, which in turn activates caspase-9 and caspase-3, leading to cell apoptosis. IH intermittent hypoxia, TNF-α tumor necrosis factor α, AP-1 activator protein-1, MAPK mitogen-activated protein kinase, NF-κB nuclear factor kappa B, C/ΕBPα CCAAT-enhancer-binding protein-α, Egr-1 early growth response protein-1, PAI-1 plasminogen activator inhibitor-1, ER endoplasmic reticulum stress, PERK protein kinase-like kinase, ATF6 transcription factor 6, IRE1 inositol requiring enzyme 1, CHOP C/EBP-homologous protein, XBP1 X-box protein-1, eIF2α e α-subunit of eukaryotic initiation factor 2

Plasminogen activator inhibitor-1 (PAI-1) levels are consistently elevated in OSAS patients,^414–416^ and there are multiple pathways through which OSAS can trigger PAI-1 upregulation. The metabolism of PAI-1 has been implicated in several diseases and conditions, including cardiovascular disease,^417^ metabolic diseases,^418^ and cancer.^419^ Cells exposed to hypoxia showed increased PAI-1 mRNA expression and stability.^420–422^ ROS are involved in most of the mechanisms regulating PAI-1 expression. Incubation of endothelial cells with H_2_O_2_ induced a significant increase in PAI-1 mRNA and protein expression.^423^ In contrast, the PAI-1 promoter is repressed by up to 75% in the presence of antioxidants.^424^ The ROS-induced increased transcription and expression of PAI-1 is mediated by activation of the MAPK and NF-κB pathways, which are tightly linked to proinflammatory pathways.^425,426^ In addition, in vitro and in vivo experimental studies as well as clinical studies, have identified TNF-α as an important factor in increasing PAI-1 expression.^427–429^ In endothelial cells, TNF-α upregulates PAI-1 levels and is abolished by N-acetylcysteine, suggesting that ROS are mediators.^424^ IL-6 is another inflammatory cytokine that regulates PAI-1 upregulation. Animals injected with IL-6 had a significant increase in PAI-1 levels, whereas the use of an IL-6 receptor antagonist decreased PAI-1 expression.^430,431^ IL-6 can also activate the MAPK/NF-κB signaling pathway, leading to increased transcription of PAI-1.^432,433^ PAI-1 is one of the major transcriptional targets of HIF-1α. Hypoxic stimulation by IH could promote HIF-1α signaling and the upregulation of PAI-1.^434^ In addition, IH-induced HIF-2α, CCAAT-enhancer-binding protein-α (C/ΕBPα) and early growth response protein-1 (Egr-1) could also upregulate PAI-1 expression^435,436^ (Fig. 6a).

Recent studies have demonstrated endoplasmic reticulum (ER) stress in the brain,^437,438^ heart,^439,440^ kidney,^441^ and liver^442^ of rodents exposed to IH. The ER is an important organelle for protein synthesis, folding, lipid biosynthesis, secretion, and cell homeostasis.^443^ When cells are stimulated by hypoxia or oxidative stress, homeostasis is disrupted.^444^ The accumulation of unfolded and misfolded proteins in the ER activates ER stress, which in turn triggers the unfolded protein response (UPR).^445^ UPR activation is regulated by the chaperone protein glucose-regulated protein BiP/GRP78.^446^ Prolonged or severe ER stress induces accelerated separation of BiP and GRP78,^439^ which activates protein kinase-like kinase (PERK), transcription factor 6 (ATF6) and inositol requiring enzyme 1 (IRE1).^443,445^ Activated ATF6, PERK, and IRE1 accelerate the activation of CHOP protein,^447^ which mediates apoptosis.^448^ CHOP deficiency protects cells from apoptosis induced by excessive ER stress.^449,450^ The UPR in mammals has three branches: the IRE1 pathway, PERK pathway, and ATF6 pathway.^451–453^ Phosphorylated IRE1 activates the downstream target proteins JNK and p38 MAPK.^454,455^ A study has shown that phosphorylation of JNK both activates proapoptotic BIM and inhibits antiapoptotic Bcl-2.^456^ In addition, the activated ATF6 pathway and PERK pathway are also involved in ER stress-related apoptosis. XBP1 is spliced by the endoribonuclease for IRE1 under ER stress,^457^ acting as a potent transcription factor for CHOP.^458^ IH in patients with OSAS increases ROS generation, which reduces the production of functional proteins and even leads to apoptosis.^446^ Several studies have confirmed that the levels of ER stress-related proteins, including JNK, MAPK, GRP78, CHOP, PERK, p-eIF2α, and ATF4, were dramatically increased when exposed to IH.^446,459^ Cai et al. found that the PERK-eIF2α signaling pathway was involved in apoptosis in rats under IH conditions.^460^ In addition, the expression of IRE1-XBP1 and ATF6 was significantly increased in rat cardiac tissues after IH exposure for 5 weeks.^439^ In another study of cardiovascular disease in rats, the protein expression of the ER stress marker proteins BiP, PERK, CHOP, and ATF4 was increased in IH.^461^ During IH, Bcl-2/Bax is low, and activation of caspase-3, caspase-9, caspase-12, and JNK is induced^439,455,462^ (Fig. 6b).

### Epigenetic alterations in OSAS

Epigenetics is generally defined as heritable phenotypic changes that do not involve DNA sequence changes that are not directly encoded by modifications of the nucleotide genomic sequence but by posttranslational modifications of DNA and histones and the regulation of noncoding RNAs.^463^ Recent studies have shown that epigenetic changes are associated with the development of OSAS and its pathogenesis, but the specific mechanisms of action are currently unknown. Below, we review relevant studies on the relationship between epigenetics and OSAS, and further understanding of the interplay between genetic and environmental factors through epigenetic regulation will be valuable to gain insight into the mechanisms underlying OSAS-associated oxidative stress, low-grade inflammation, and sympathetic hyperactivity.

Noncoding RNAs include microRNAs (miRNAs) and long noncoding RNAs (lncRNAs).^464^ MiRNAs, a class of single-stranded RNAs consisting of 19 to 25 nucleotides in length, can regulate gene expression by binding to mRNA. MiRNAs can mediate posttranslational gene silencing and thus negatively regulate target genes.^465–467^ Recent studies have found that multiple miRNAs can influence the IH process and influence hypoxia-induced apoptosis.^468^ For example, in a rat model, miR-26b-5p upregulation and miR-207 downregulation were involved in IH-induced cognitive impairment by increasing Bax and cleaved caspase-3 expression and reducing Bcl-2 expression in the hippocampus.^469^ MiR-155 promoted oxidation and enhanced the IH-induced NLRP3 inflammasome pathway by repressing the target forkhead box protein O3 (FOXO3a) gene in a murine model and HK-2 cells. Interestingly, IH-induced NLRP3 inflammasome activation in renal tubular cells was then suppressed by inhibiting miR-155 expression.^470^ In addition, miR-155 has been shown to have a proapoptotic function in diseases where other antiapoptotic proteins, such as clusterin, are decreased and correlate with increased clusterin levels in OSAS.^471,472^ MiR-664a-3p is downregulated in patients with OSAS and is negatively correlated with AHI and carotid intima-media maximum thickness, suggesting that circulating miR-664a-3p has the potential to serve as a noninvasive marker of atherosclerosis in OSAS.^473^ MiRNAs have been considered ideal biomarkers in the era of precision medicine, and sequencing analysis has shown that the expression levels of miR-199-3p, 107, and 485-5p were downregulated, whereas the expression level of miR-574-5p was upregulated in OSAS patients, suggesting that the differentially expressed miRNAs are closely related to OSAS.^474^ Based on the current study, miRNAs could be potential indicators for the diagnosis and treatment of OSAS in the future (Table 4).

**Table 4:** Main studies on microRNAs in obstructive sleep apnea syndrome (OSAS)/intermittent hypoxia (IH)

MiRNA name	Expression in IH	Target gene	Original source	Quantification approach	Main findings	Reference
miR-26b-5p	Up	Unknown	Rat hippocampus	miRNA microarray and qRT‒PCR	miR-26b-5p and miR-207 could be involved in cognitive impairments	Gao et al. (2017)^469^
miR-207	Down					
miR-155	Up	*FOXO3a*	Renal tissue and HK‐2 cells	RT‒qPCR	miR‐155 might be a positive regulator of the NLRP3 pathway to enhance renal injury	Wu et al. (2018)^470^
miR-664a-3p	Down	Unknown	Serum of OSAS patients	qRT‒PCR	Negative correlation of miR-664a-3p expression with AHI and maximum carotid intima-media thickness (CIMT) and positive correlation with the lowest oxygen saturation (LOS); miR-664a-3p as a candidate biomarker of atherosclerosis in OSAS	Li et al. (2018)^473^
miR-199-3p	Down	Unknown	Serum of OSAS patients	LNA oligonucleotide microarrays and qRT‒PCR	Involved in hypoxia, metabolism, and oxidative stress	Li et al. (2017)^474^
miR-107						
miR-485-5p	Up					
miR-574-5p						
miR-130a	Up	*GAX*	Blood of OSAS patients; human umbilical vein endothelial cells	qRT‒PCR	miR-130a may contribute to the development of OSAS-associated pulmonary hypertension by downregulating the expression of *GAX*	An et al. (2017)^684^
miR-365	Down	*IL-6*	Hepatocyte, stellate cell, and macrophage cell lines; serum of OSAS patients	qRT‒PCR	miR-365 acts as an important trigger for the production of proinflammatory cytokines and activation of macrophages in OSAS patients	Schaefer et al. (2017)^685^
miR-185	Down	*CoLA1*	Lung tissue of dogs; COPD lung tissue; human primary pulmonary cells	qRT‒PCR	OSAS could inhibit miR-185 and promote *CoLA1* expression leading to lung remodeling	Ding et al. (2016)^686^
miR-34a-5p	Up	*Bcl-2*	Human coronary artery endothelial cells	qRT‒PCR	miR‐34a‐5p activated beclin-1 through *Bcl‐2* inhibition in IH and participated in IH-induced endothelial cell autophagy	Lv et al. (2019)^687^
miR-630	Down	*Nrf2, AMP kinase, and tight junction pathways*	Plasma of pediatric OSAS patients and human microvascular endothelial cells	miRNA microarrays and qRT‒PCR	miRNA-630 as a putative key mediator of endothelial dysfunction in children with underlying OSAS	Khalyfa et al. (2019)^688^
miR-145	Down	*Smad3*	Canines; human aortic tissue; vascular smooth muscle cells from rats	qRT‒PCR	OSAS could activate the miR-145/*Smad3* signaling pathway to promote aortic fibrosis, apoptosis and sympathetic nerve sprouting, which cause aortic structural and autonomic remodeling	Yu et al. (2017)^689^
miR-146a-5p	Up	*XIAP*	H9c2 cells	qRT‒PCR	miR-146a-5p could aggravate IH-induced H9c2 cell injury by attenuating H9c2 viability and promoting its apoptosis by targeting *XIAP*	Lin et al. (2019)^690^
miR-30a	Up	*Beclin-1*	Mouse endothelial cells	RT‒qPCR	Upregulated miR-30a significantly reduced beclin-1 levels to attenuate endothelial cell autophagy in vitro and in vivo, which aggravated IH-induced endothelial cell injury	Bi et al. (2019)^691^
miR-31	Up	*PKCε*	H9c2 neonatal cardiomyocytes	qRT‒PCR	Upregulation of miR-31 decreased the mRNA and protein expression of *PKCε* to promote myocardial hypertrophy	Ren et al. (2018)^692^
miR-224-5p	Down	*NLRP3*	Mouse brain tissues and microglial BV2 mouse cells	qRT‒PCR	miR-224-5p reduces microglial inflammatory activation by regulating *NLRP3* expression	Du et al. (2020)^481^
miR-218	Up	*Robo1*	Mice aortic endothelial cells	qRT‒PCR	Upregulated expression of miR-218 promotes IH-induced apoptosis in aortic endothelial cells targeting *Robo1*	Liu et al. (2017)^693^
miR-203	Down	*SELENOP HIP/PAP*	Human hepatocytes	qRT‒PCR	IH upregulated the levels of *SELENOP* in human hepatocytes to potentiate insulin resistance and upregulated the levels of *HIP/PAP* mRNAs to promote cell proliferation via a miR-203-mediated mechanism.	Uchiyama et al. (2017)^694^
miR-452	Down	*RETN, TNF-α, and CCL2*	Mouse adipocytes and human liposarcoma adipocytes	qRT‒PCR	IH downregulated miR-452, resulting in increased levels of *RETN*, *TNFα*, and *CCL2*, leading to insulin resistance	Uchiyama et al. (2019)^695^
miR-126a-3p	Down	*HIF-1a*	The blood, heart tissues, and abdominal aortas of rats; rat aortic smooth muscle cells	qRT‒PCR	IH decreased miR-126a-3p levels and increased *HIF-1α* expression, which promoted hypertension in the OSAS rat model	He et al. (2020)^696^
miR-320b	Down	*USP37*	Lung cancer tissues and lung cancer cells	qRT‒PCR	IH-induced miR-320b downregulation promoted the proliferation and invasion capabilities of lung cancer cells through a *USP37*-mediated mechanism	Li et al. (2021)^697^
miR-21	Up	*Spry1/ERK/MMP-9, PTEN/PI3K/AKT and NF-κB pathways*	Rat atrial tissues	RT‒qPCR	IH-induced upregulation of miR-21 expression promotes atrial remodeling and fibrosis	Zhang et al. (2018)^698^
miR-214-3p	Up	*CTRP9*	Cardiac tissue of IH mice	qRT‒PCR	Myocardial infarction + IH upregulated miR-214-3p, inhibited cardiac *CTRP9* expression and exacerbated cardiac remodeling and heart failure	Du et al. (2020)^699^
miR-1249 miR-193 miR-218 miR-30B	Up	Unknown	Mouse aortic endothelial cell	qRT‒PCR	Different miRNA expression patterns could be induced by IH, in which downregulation of miR-193 was associated with the expression of autophagy- and apoptosis-related genes.	Liu et al. (2018)^468^
miR-16 miR-718	Down					

LncRNAs are composed of RNA strands longer than 200 nucleotides that are not translated into proteins, and experimental evidence has shown that they can regulate gene expression through a variety of mechanisms, including transcriptional activation or repression, chromatin modification, and posttranscriptional regulation.^475,476^ A microarray study of cardiac samples from rats exposed to IH for 8 weeks identified 157 lncRNAs with upregulated expression and 132 lncRNAs with downregulated expression. Three of the downregulated lncRNAs (XR_600374, XR_590196, and XR_597099) and three of the upregulated lncRNAs (XR_596701, XR_344474, and ENSRNOT00000065561) were validated by quantitative reverse transcription polymerase chain reaction. This study provides novel insights into lncRNAs in the pathogenesis of IH.^477^ Another study found that overexpressing lncRNA CPS1-IT decreased IL-1β through the transcriptional activity of HIF-1 expression to reduce pulmonary arterial hypertension in OSAS patients.^478^ Multiple studies have confirmed that the abnormal expression of lncRNAs promotes the occurrence and development of diseases, and some lncRNAs have been identified as biomarkers for diseases.^479^ LncRNA is not only a repressive regulator but also a source of miRNAs.^480^ Du et al. found that blocking the lncRNA MALAT1/miR-224-5p/NLRP3 axis suppressed hippocampal inflammation in type 2 diabetes mellitus patients with OSAS.^481^ Another experiment on aortic endothelial dysfunction in OSAS patients showed that the lncRNA maternally expressed gene 3 (MEG3) altered HIF-1α expression by competitively binding to miR-135a, and silencing MEG3 could inhibit aortic endothelial cell apoptosis and injury.^482^ More details are given in Table 5. Further studies are needed to clarify the role of lncRNAs as potential biomarkers in OSAS.

**Table 5:** Main studies on lncRNA in obstructive sleep apnea syndrome (OSAS)/intermittent hypoxia (IH)

LncRNA name	Expression in IH	Target	Original source	Quantification approach	Main findings	Reference
XR_600374 XR_590196 XR_597099	Down	Unknown	Heart samples of rats	lncRNA microarray and qRT‒PCR	This study revealed for the first time that OSAS changed the expression profile of lncRNA in the rat heart, which could help us to establish the knowledge base of cardiovascular disease pathogenesis induced by OSAS	Chen et al. (2019)^477^
XR_596701 XR_344474 ENSRNOT00000065561	Up					
CPS1-IT	Down	*HIF-1*	Pulmonary artery tissues of rats	RT‒qPCR	Decreased CPS1-IT could enhance the transcriptional activity of *HIF-1*, enhance the expression of IL-1β through the NF-κB signaling pathway, and promote pulmonary arterial hypertension in OSAS	Zhang et al. (2019)^478^
MALAT1	Up	*miR-224-5p*	Mouse brain tissues and the microglial BV2 mouse cell line	qRT‒PCR	IH increased the expression of MALAT1, further inhibited the expression of *miR-224-5p*, and finally regulated the NLRP3/IL-1β pathway and promoted hippocampal inflammation	Du et al. (2020)^481^
MEG3	Up	*miR135a*	Aortic endothelial tissues of mice	RT‒qPCR	IH induced increased expression of MEG3, and targeted *miR-135a* upregulated HIF-1α to promote aortic endothelial injury and apoptosis in IH mice	Ding et al. (2020)^482^
ROR	Up	*miR-145*	HK-2 cells	qRT‒PCR	ROR alleviated CoCl2-induced hypoxia injury through the regulation of *miR-145*	Ge et al. (2019)^700^
ENST00000592016	Up	Unknown	Plasma exosomes of OSAS patients	qRT‒PCR	The level of ENST00000592016 is correlated with the severity of OSAS and can be used as a diagnostic marker for OSAS	Chen et al. (2022)^701^
MRPL20-AS1	Down	Unknown	Male human coronary artery cells	qRT‒PCR	MRPL20-AS1 might serve as a useful tool to identify patients with severe OSAS	Zietzer et al. (2022)^702^
NONMMUT032513	Up	ZEB1 and smad5; Cmbl and ADH5 (unverified)	Mouse heart tissues	Microarray and qRT‒PCR	LncRNAs might be responsible for myocardial infarction aggravation under OSAS	Hu et al. (2021)^703^
NONMMUT074571		ZEB1 and Smtn; Cmbl and Pfdn6 (unverified)				
XIST	Up	GRα	The adenoids of patients with OSAS and NP69 cells	qRT‒PCR	XIST reduces the expression of GRα through the NF-κB-dependent signaling pathway, thereby promoting the occurrence and development of OSAS	Zhou et al. (2021)^704^
XR_595552	Up	PI3K/AKT pathway	H9c2 cells	qRT‒PCR	XR_595552 may play a protective role in alleviating IH-induced cardiomyocyte injury by regulating the PI3K/AKT pathway	Chen et al. (2023)^705^

DNA methylation, the best-known and best-characterized epigenetic modification, is a heritable, reversible epigenetic change that mediates the transcriptional silencing of genes by altering transcription factors in the promoter regions of genes and activates gene transcription by alternative splicing.^483^ DNA hypermethylation usually leads to transcriptional repression and decreased gene expression, whereas DNA hypomethylation affects chromosomal stability.^484^ Currently, there are few studies on the role of DNA methylation in OSAS. A previous study showed that the FOXP3 gene, which regulates T regulatory lymphocyte expression, showed increased DNA methylation in a total cohort of children with OSAS who had increased systemic inflammatory responses, suggesting that epigenetic-mediated downregulation of T regulatory lymphocytes might be an important determinant of OSAS-induced systemic low-grade inflammation.^485^ IH-exposed neonatal rats exhibit increased DNA methylation in the promoter region of the superoxide dismutase (SOD2) gene, and methylation modification has long-lasting effects on elevated chemoreflex sensitivity and hypertension in adult rats.^486^ Another study also confirmed that the impairment of respiratory and carotid body chemosensory reflexes by IH is partly the result of inhibition of antioxidant enzyme (AOE) genes via DNA methylation, including peroxiredoxin 4 (Prdx4) and thioredoxin reductase (Txnrd2).^487^ Previously, in a study of epigenomic DNA methylation, Chen et al. demonstrated multiple differentially methylated genes associated with OSAS and its adverse outcomes. Studies have found that hypomethylated interleukin 1 receptor 2 (IL-1 R2) and hypermethylated androgen receptor (AR) may be important contributors to disease severity, whereas hypomethylated natriuretic peptide receptor 2 (NPR2) and hypermethylated speckled protein 140 (SP140) may be biomarkers that predispose patients with OSAS to excessive daytime sleepiness^488^ (Table 6).

**Table 6:** Main studies on DNA methylation in obstructive sleep apnea syndrome (OSAS)/intermittent hypoxia (IH)

Gene name	Methylation level	Original source	Main findings	Reference
FOXP3	Hyper	Blood of OSAS children with and without high hsCRP	In OSAS children with increased systemic inflammatory response, methylation of the FOXP3 gene is more likely to increase, which may provide potential biomarkers for terminal organ susceptibility	Kim et al. (2023)^485^
AOEs	Hyper	Carotid body and adrenal medulla of rats exposed to IH	The persistent cardiopulmonary abnormality caused by IH is due to the long-term inhibition of the AOE gene by DNA methylation, resulting in a continuous increase in ROS levels in the carotid body chemosensory reflex pathway	Nanduri et al. (2017)^487^
IL1R2	Hypo	Blood of sleep-disordered breathing (SDB) patients with ODI >30 and SDB patients with ODI ≤30	IL1R2 hypomethylation and AR hypermethylation might be important determinants of disease severity	Chen et al. (2016)^488^
AR	Hyper			
NPR2	Hypo	Blood of SDB patients with excessive daytime sleepiness (EDS) and SDB patients without EDS	NPR2 hypomethylation and SP140 hypermethylation might be biomarkers of EDS in patients with OSAS.	
SP140	Hyper			
eNOS	Hyper	Blood of OSAS children	Endothelial dysfunction caused by eNOS hypermethylation	Kheirandish-Gozal et al. (2016)^706^
Ace1 Agt	Hypo	CD31+ endothelial cells isolated from the mesenteric arteries of IH-exposed mice	IH-exposed mice had higher DNA methylation levels of Ace1 and Agt genes, which led to persistent changes in the renin-angiotensin system regulation and endothelial function, eventually leading to hypertension	Chu et al. (2015)^707^
FPR1	Hypo	Blood leukocyte of OSAS patients	Aberrant DNA methylation of the FPR1/2/3 gene in OSAS patients may be involved in the severity of the disease and the occurrence of diabetes mellitus or cardiovascular disease.	Chen et al. (2020)^708^
FPR2	Hyper			
FPR3				

## Diseases associated with OSAS

Repeated processes of airway collapse and obstruction caused by various pathological factors in OSAS patients lead to recurrent apnea and periodic arousal during sleep, which eventually cause IH and sleep fragmentation. These core factors stimulate cell and molecular mechanisms, including increased sympathetic nerve activity, metabolic dysregulation, systemic inflammation, oxidative stress, and endothelial dysfunction, which have been identified as pathogenic in different clinical and experimental models and could lead to various OSAS-related complications. Different mechanisms may predominate in specific comorbidities, and the evidence for an independent association between OSAS and comorbidities is stronger for some comorbidities than others. While the detailed molecular mechanisms leading to the development of cardiovascular, cerebrovascular, and other diseases in OSAS are complex and several different mechanisms are involved, it seems that oxidative stress and inflammation are fundamental underlying mechanisms and are closely related to diseases in various systems throughout the body.

### OSAS and cardiocerebrovascular disorders

A large body of evidence indicates that OSAS is associated with a number of cardiovascular complications,^1,19,489,490^ including systemic hypertension, arrhythmias, coronary artery disease, and stroke. The most convincing epidemiologic evidence of a causal relationship between OSAS and hypertension was provided in the 4-year follow-up results from the Wisconsin Sleep Cohort study.^491^ It is estimated that approximately 50% of patients with OSAS suffer from hypertension, and 30–40% of patients with hypertension suffer from OSAS.^492,493^ This is particularly true in patients with resistant hypertension, of whom up to 80% may suffer from OSAS. The Sleep Heart Health Study (*n* = 6132) also showed an increased likelihood of hypertension with increasing severity of OSAS, and the prevalence of hypertension was 59, 62, and 67% in patients with mild, moderate, and severe sleep apnea, respectively.^494^ In addition, OSAS is also responsible for masked hypertension in many cases.^19,491^ The ROS-dependent increase in sympathetic nerve activity (SNA) is a prominent feature of OSAS and has been shown to be associated with OSAS-related atrial fibrillation (AF), heart failure, and hypertension.^19,386,495^ Sympathetic outflow to the kidney is increased and stimulates renin release, which leads to increased circulating levels of angiotensin II and aldosterone, which in turn increases vascular resistance to constrict the vessels and raise blood pressure.^496^ Circulating and urinary catecholamines, which are biomarkers of elevated SNA, are also elevated in patients with OSAS.^148^ Emerging evidence implicates transcriptional changes by HIF-1α as an important molecular mechanism by which IH leads to SNA and hypertension.^148^ Animal studies of OSAS have shown activation of HIF-1α in myocardial tissue and increased expression of its downstream gene endothelin. Endothelin is a potent vasoconstrictor that causes blood pressure elevation.^497^ Advances in the understanding of cardiovascular disease in OSAS are closely related to the understanding of the development of coronary artery disease, but the underlying mechanisms remain poorly understood. The pathogenesis is likely to be a multifactorial process involving several mechanisms, including SNA, oxidative stress, vascular smooth muscle cell activation, lymphocyte activation, increased lipid levels, and lipid peroxidation within macrophages leading to endothelial dysfunction, which largely contributes to the development of various cardiovascular diseases, particularly atherosclerosis.^407^ IH triggers a molecular response that generates inflammation and oxidative stress and induces the formation of ROS, which in turn activates the inflammatory cascade by activating the transcription factor NF-κB and downstream genes such as inflammatory cytokines and adhesion molecules.^2,386^ Various activated blood cells produce more ROS, adhesion molecules, and proinflammatory cytokines. Adhesion molecules promote the accumulation of platelets, leukocytes, and possibly red blood cells on the vascular endothelium.^379^ Clinical studies have confirmed that blood cells from patients with OSAS present a proinflammatory and prothrombotic phenotype; additionally, the role of monocytes in the initiation and propagation of the progression of atherosclerosis is well established, and resident or circulating leukocytes mediate monocyte adhesion to the endothelium, which might promote thrombosis, endothelial dysfunction, and atherosclerosis.^498–502^ Growing evidence indicates a concomitant prevalence of AF of 21–74% in patients with OSAS,^503^ suggesting that OSAS might be a causative factor in AF pathogenesis.^504^ A potential explanation is the enhanced sympathetic and vagal nerve activities caused by hypoxemia, which triggers AF during acute OSAS.^505^ Chronic recurrence and sudden negative changes in intrathoracic pressure play a crucial role in atrial autonomic, structural, and electrical remodeling, leading to structural and functional atrial remodeling that triggers AF by contributing to atrial fibrosis.^19,506^ Multiple prospective studies have demonstrated a strong association between moderate-severe OSAS and stroke. The Wisconsin Sleep Cohort study found that an AHI >20 was significantly associated with an increased risk of stroke,^507^ while another study found that men with an AHI >15 had a threefold increased risk of stroke.^508^ Unsurprisingly, concurrent AF substantially increased the risk of stroke in patients with OSAS. Continuous positive airway pressure (CPAP) therapy has been shown to benefit the incidence and recurrence of stroke in patients with OSAS,^509^ and another study showed that CPAP therapy can reduce the rates of stroke and cardiovascular events in patients with severe OSAS.^510^ Hypertension or other traditional vascular risk factors do not fully explain the association of OSAS with stroke, and the underlying mechanisms include multiple factors such as hypercoagulability, cardiac arrhythmias, inflammation, oxidative stress, dysautonomia, and dyslipidemia.^19^

Accumulating evidence suggests that oxidative stress, inflammation, and molecular mechanisms play an important role in the pathophysiology of cardiocerebrovascular disease in patients with OSAS. In addition, a clinical lesson learned from understanding the underlying pathophysiology of OSAS with the accompanying comorbidities is that to prevent cardiovascular morbidity, treatment of breathing disorders during sleep might need to start at the earliest possible age.

### OSAS and neurological disorders

Prolonged periods of IH in patients with OSAS could impact multiple CNS systems, all of which ultimately lead to severe neurocognitive and behavioral deficits, including a decline in cognitive functions, such as memory, executive function and comprehension, mood disturbances, insomnia, and/or excessive daytime sleepiness. In addition, OSAS may promote the development of neurodegenerative diseases.^511,512^ The results of animal studies from our team have shown that IH induces severe neuronal injury (especially in the hippocampal CA1 region), enhances inflammation, and activates astrocytes in the rat brain. The rats in the IH group showed a much longer escape latency when locating the hidden platform and much less time spent in the target quadrant than the normal control group. In addition, we found that IH significantly increased ROS levels, decreased manganese superoxide dismutase (Mn-SOD) and catalase (CAT) expression, increased the levels of lipid peroxidation products [including malondialdehyde (MDA) and DNA damage products, such as 8-hydroxy-2’-deoxyguanosine (8-OHdG)] in the hippocampus and significantly increased caspase-1, IL-1β, and IL-18 expression in the frontal medial cortex in mice.^513,514^ IH-induced increases in neuroinflammation, oxidative stress, and brain tissue damage in mice might account for the diminished performance in the Morris water maze test. We used the Montreal Cognitive Assessment (MoCA) and Epworth Sleepiness Scale to evaluate the cognitive status of OSAS patients in our previous clinical study. The findings showed significant impairments in attention, delayed memory function, and executive function in patients with OSAS, and the MoCA scores were negatively correlated with the AHI and oxygen desaturation index and positively correlated with the lowest oxygen saturation. In this study, we compared the automatic processing of emotional facial expression patterns between OSAS patients and matched normal controls by evaluating expression-related mismatch negativity (a brain electrophysiological detection tool) and found that OSAS patients suffer from cognitive impairment in the automatic processing of emotional facial expressions under the preattentive condition.^21^ Structural and functional alterations in brain anatomy and function in OSAS patients provide indirect evidence that OSAS causes damage to brain structures over time. Perhaps these changes underlie cognitive impairment. Studies have suggested a decrease in gray matter in the prefrontal cortex, anterior cingulate cortex, thalamus, parietal cortex, parahippocampal gyrus, inferior temporal gyrus, hippocampus, and cerebellum in patients with OSAS.^511,515^

It is well known that the brain is more sensitive to hypoxia than other organs and requires more energy and oxygen consumption. Clinical and animal findings suggest that IH resulting from OSAS can lead to structural neuronal damage and dysfunction in the CNS, with oxidative stress and inflammatory damage being the pathophysiological basis.^516^ Accumulating evidence supports the view that, in the CNS, IH may induce ROS production in the CNS, oxidative stress overactivation, and inflammatory damage leading to neuronal apoptosis and/or necrosis that, in turn, contributes to the development of OSAS-related cognitive impairments.^517^ Brain tissue NF-κB, TNF-α, CRP, IL-1β, IL-6, and cyclooxygenase-2 (COX-2) levels were measured in IH animal models, which were consistent with the changes seen in human plasma. The standardized regression test showed significant associations between proinflammatory cytokines and neurocognitive performance.^516^ A recent study confirmed that nocturnal overactivation of the sympathetic nervous system can lead to visuospatial dysfunction in patients with OSAS.^518^ The most prominent maladaptive effect of IH is neuroinflammation, and although the exact neural cell source of the associated processes is still not fully understood, microglial activation may be important. The findings showed that IH exposure resulted in a significant increase in microglial activity and hippocampal neuronal apoptosis, as well as increased levels of related inflammatory markers (NF-κB, TNF-α, and IL-1β).^519^ Microglia, the major inflammatory cells of the CNS, mediates oxidative stress and inflammation through mitochondria, NADPH oxidase, and the release of excitotoxic neurotransmitters. Recently, we demonstrated an important role for microglia in the hippocampus in the development of diabetic encephalopathy by single-cell RNA sequencing.^520^ NADPH oxidase is involved in microglia-mediated neurotoxicity and microglial activation. Activated microglia express high levels of inducible nitric oxide synthase (iNOS) and COX-2 isoforms, ultimately leading to increased ROS generation. Furthermore, activated microglia trigger the NF-κB signaling pathway, which regulates the immune inflammatory response, oxidative stress, and memory. Studies have confirmed that this pathway plays an important role in hypoxia.^521^ JNK is a member of the MAPK family and has a complex relationship with the NF-κB pathway. IH effectively activated the NF-κB/JNK pathway and its downstream signaling molecules, confirming the role of the NF-κB-mediated JNK pathway in hippocampal injury and cognitive dysfunction in IH model rats.^522^ p38 MAPK is also a member of the MAPK family, and its activation has adverse effects on learning and memory. In an IH animal model, p38 MAPK levels were significantly increased, which could activate the NF-κB signaling pathway, releasing cytokines such as IL-1 β, IL-6, and TNF-α, oxidative species, and adhesion molecules.^523^ The release of cytokines, in turn, promotes the production of ROS by microglia, thereby perpetuating inflammation and aggravating ongoing oxidative stress.^524,525^ CNS neuronal damage and apoptosis from IH might involve other mechanisms. For example, brain-derived neurotrophic factor (BDNF), an important neuromodulator of CNS function, significantly prevents oxidative stress-induced neuronal damage in the CNS.^526^ In addition, microglia release excitatory toxic neurotransmitters, such as glutamate, and studies have shown that higher glutamate concentrations are found in the cerebral cortex of OSAS patients, leading to excitotoxicity-induced neuronal dysfunction and apoptosis.^527^

**Fig. 7:**
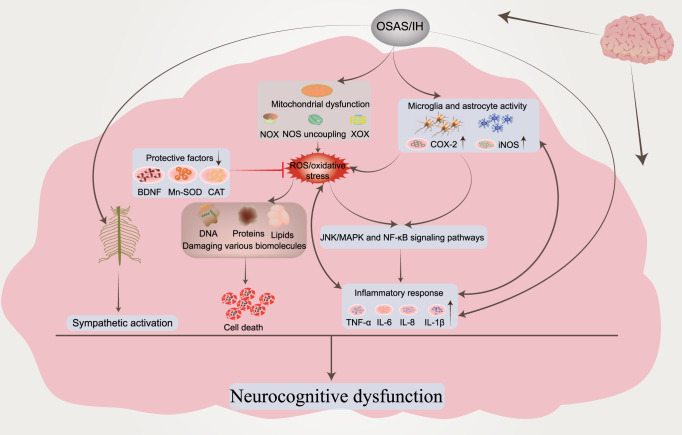
Proposed interactions between neurological disorders and other pathological processes induced by OSAS/IH-induced elevated ROS levels. OSAS/IH upregulates the expression of ROS in the brain, and the inhibitory effect of protective neurotrophic factors on ROS is weakened, which further leads to an increase in ROS. The macromolecular substances in injured nerve cells cause nerve cell death and activate inflammation-related signaling pathways to release inflammatory factors. Sympathetic nerve activation by OSAS/IH could cause cognitive impairment independently of other mechanisms. In addition, OSAS/IH can directly activate microglia and astrocytes and promote the release of inflammatory cytokines in the central nervous system. Excessive neuroinflammatory responses could, in turn, promote the activation of glial cells, resulting in synaptic damage and loss, neuronal necrosis, and apoptosis and ultimately leading to exaggerated neurocognitive dysfunction. BDNF brain-derived neurotrophic factor, Mn-SOD superoxide dismutase, CAT catalase, COX-2 cyclooxygenase-2, iNOS inducible nitric oxide synthase

Undoubtedly, most OSAS patients develop cognitive and neurologic dysfunction. Furthermore, these findings suggest a strong link between inflammation and cognitive impairment in OSAS (Fig. 7). At the same time, evidence regarding its links with neurological diseases is similarly accumulating. The evidence for its links with major psychiatric and neurologic disorders is similarly accumulating. However, the exact nature of the mechanisms responsible for these effects remains to be determined and must be investigated further.

### OSAS and metabolic diseases

Growing evidence in animal models of OSAS suggests that IH is independently associated with metabolic dysfunction. In particular, OSAS was independently associated with insulin resistance, suggesting that OSAS might be an important factor in the development of type 2 diabetes and so-called metabolic syndrome (MS), namely, obesity, insulin resistance, hypertension, and dyslipidemia. Studies have confirmed that the levels of fasting blood glucose and insulin resistance in OSAS patients are significantly higher than those in non-OSAS patients, and the severity of OSAS is related to an increase in insulin resistance. Moreover, the relationship between OSAS and insulin resistance also applies to nonobese patients.^528^ In addition, clinical data suggest that the AHI is an independent risk factor for insulin resistance and type 2 diabetes. With each unit increase in the AHI, the level of insulin resistance increased by 0.5%.^529,530^ In vivo kinetic studies of glucose metabolism have also demonstrated that severe OSAS impairs insulin sensitivity, glucose effectiveness, and pancreatic β-cell function.^531^ Oxidative stress and inflammation induced by intermittent hypoxemia in patients with OSAS may be key factors in insulin resistance. Inflammatory factors induced by OSAS, including TNF-α, IL-6, and IL-18, which activate NF-κB, JNK, and other downstream signaling pathways, inhibit insulin receptors and the phosphorylation of insulin receptor substrates, leading to insulin resistance.^532^ IH decreases glucose uptake in muscle, increases β-cell proliferation and β-cell death^1^ and can also affect ATP synthesis in pancreatic islet β cells, thereby inhibiting insulin secretion.^532^ Increased sympathetic tone in OSAS patients is a key mediator of deterioration of glycemic and insulin homeostasis, and increased levels of catecholamines after arousal directly stimulate glycogen mobilization and inhibit muscle glucose uptake, stimulate glucagon secretion, and inhibit insulin secretion.^533^ In addition, IH has been shown to induce lipid abnormalities, such as increased total cholesterol, triglycerides, high-density lipoprotein-cholesterol (HDL-C), very-low-density lipoprotein (VLDL), and low-density lipoprotein (LDL) levels, and the severity of lipid elevation is proportional to the severity of hypoxic stimulation.^532^ Several cross-sectional studies have shown that OSAS is independently associated with increased levels of total cholesterol, LDL, and triglycerides and that treatment of OSAS with CPAP may have beneficial effects on the lipid profile.^532,534,535^ In addition to the promotion of SREBP expression by IH mentioned earlier, IH is also related to lipoprotein lipase inhibition in adipose tissue, which leads to an increase in plasma chylomicron particles and VLDL that may be conducive to the progression of atherosclerosis.^536^ IH increases leptin gene expression levels, acting centrally and peripherally to inhibit insulin secretion while increasing glucose uptake. A number of reports have demonstrated that serum leptin levels are positively correlated with AHI and hypoxemia in patients with OSAS. The higher the serum leptin level is, the higher the AHI and the longer the duration of hypoxemia.^532,537^ Conversely, adiponectin’s effects counter those of leptin, an insulin-sensitizing hormone with antiatherogenic, anti-inflammatory, and antidiabetic effects, and IH may inhibit adiponectin secretion; studies have demonstrated significantly lower circulating adiponectin levels in patients with OSAS and a negative correlation with the AHI.^538,539^

**Fig. 8:**
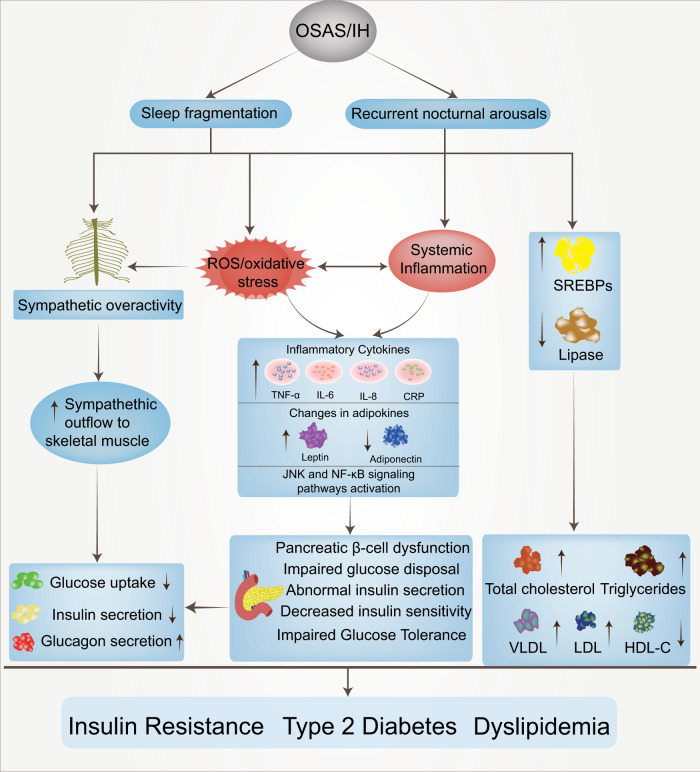
Theoretical framework of possible mechanisms by which sleep fragmentation and recurrent nocturnal arousals might contribute to the occurrence of metabolic syndrome. Two key features of OSAS, namely, sleep fragmentation and recurrent nocturnal arousal, could lead to increased sympathetic nerve activity and altered glucose metabolism in skeletal muscle. ROS production in fat and activation of inflammatory pathways lead to the increased release of inflammatory factors and changes in fat-related factors, leading to metabolic dysfunction and impaired islet function. In addition, elevated SREBPs and decreased lipase caused by inflammation and oxidative stress lead to associated lipid/lipoprotein abnormalities

In summary, OSAS leads to metabolic dysfunction (Fig. 8). However, the exact relationship between OSAS and metabolic diseases remains controversial, and most cross-sectional studies lack adequate sample sizes. The specific mechanism remains to be further studied. In addition, there is an urgent need to increase awareness of their strong association, and early detection of comorbidities cannot be overemphasized.

### OSAS and cancer

Over the past years, circumstantial, epidemiological, clinical, and animal-based experimental evidence has provided significant support that OSAS affects tumorigenesis and tumor development. A large multicenter cohort of cancer-free patients with OSAS showed that nocturnal hypoxemia was associated with all-cancer incidence in OSAS patients.^540^ Patients younger than 45 years with severe OSAS have a significantly higher incidence of all types of cancer than the general population.^541^ Epidemiologic studies have also confirmed that OSAS is associated with increased cancer-related mortality. A dose‒response relationship between OSAS severity and cancer-specific mortality was observed over a 22-year follow-up of 1522 participants in the community-based Wisconsin Sleep Cohort study, with severe OSAS conferring a nearly fivefold risk of death from cancer.^542^ OSAS appears to elevate the incidence of some tumor types, including lung cancer, breast cancer, prostate cancer, nasopharyngeal tumors, and melanoma. In certain types of tumors, IH exposure that mimics the oxygenation pattern induced by OSAS during sleep promotes the growth, invasion, and metastasis of lung cancer, colon cancer, and melanoma.^543^

OSAS-associated intermittent hypoxemia may affect tumor biology via several mechanisms, including oxygen-sensing pathways, chronic systemic inflammation, oxidative stress, endothelial dysfunction, and immune dysregulation. The carotid body response to hypoxemia and sleep fragmentation increases sympathetic nervous system activity, which might affect the tumor and its microenvironment and contribute to cancer progression.^147^ Oxidative stress promotes tumor occurrence and progression, and it has been mentioned previously that increased oxidative stress can cause damage to DNA, proteins, and lipids, leading to gene mutations, altered cell growth patterns, and, ultimately tumorigenesis. It has also been demonstrated that in sleep apnea, oxidative stress-induced DNA damage can increase the probability of genetic mutations and hence increase cell malignant transformation potential.^544^ In addition, ROS activate the AP-1 and NF-κB signaling pathways,^545^ with increased levels of AP-1 observed in many human tumor types. AP-1 regulates the expression of cell cycle regulators (p53, p19, p21, and cyclin D1) while also affecting the downregulation of tumor suppressor genes, thereby inducing hyperproliferation and tumorigenesis. NF-κB can induce the expression of cell proliferation molecules, apoptosis inhibitor factors, proangiogenic factors, and enzymes involved in extracellular matrix degradation. The activation of NF-κB increases the expression of genes associated with the inflammatory response and increases the cellular response to proinflammatory factors. In particular, the expression of COX-2, CC motif chemokine ligand 2 (CCL2), CXC motif chemokine ligand (CXCL)1, IL-8, and IL-6 was increased. All are inflammatory mediators involved in various neoplastic processes.^546^ Thus, NF-κB is regarded as having an important role in tumor development. ROS generated by IH can also activate HIF-1α, which is highly expressed in many solid tumors and plays an important role in many aspects of tumor angiogenesis, cell survival, proliferation, apoptosis, metastasis, invasion, and metabolism.^547^ Moreover, IH can affect the expression of HIF-1α downstream genes by upregulating the transcription of HIF-1α, for example, upregulating the expression of the vascular endothelial growth factor gene (VEGF), which in turn induces tumor angiogenesis and promotes tumor development, as also demonstrated in animal experiments using IH (or intermittent blood flow).^548^ Downregulation of immune responses against cancer is an important mechanism by which IH might affect tumor growth and aggressiveness. Data from studies of tumor-specific immune function in patients with OSAS also suggest that IH might contribute to reduced innate antitumor responses. The upregulation of tumor-promoting gene sets in untreated patients with severe OSAS was demonstrated by genome sequencing in circulating leukocytes, and the expression of these genes was downregulated after approximately one month of CPAP treatment.^549^ A key effector cell in cancer biology is the macrophage, and tumor-associated macrophages (TAMs) have now been identified as a crucial component of the cancer microenvironment, especially those with an anti-inflammatory M2 phenotype, inhibiting the antitumor activity of T cells and NK cells and releasing growth factors, cytokines, inflammatory mediators, and proteolytic enzymes involved in tumor growth and invasion to promote their proliferative development.^550^ Animal model experiments have found that IH exposure selectively induced a tumor-promoting phenotype, and TAMs explanted from IH-exposed mice enhanced the proliferation and invasiveness of lung epithelial cancer cells in vitro.^551^ More specifically, IH recruits more TAMs to participate in tumor progression and accelerates their transformation from an antitumor phenotype (M1) to a tumor-promoting phenotype (M2). It is interesting to find that CCL2 is a TAM recruiting factor,^552^ and PGE2 has an effect against tumor cells, playing an important role in the mechanism of cancer immune evasion. PGE2 inhibits the anticancer function of NK cells and enhances the cancer-promoting function of M2 macrophages and regulatory T (Treg) cells.^553^ Increased sympathetic activity caused by apnea may also contribute to cancer development. In vitro studies have shown that adrenergic signaling can regulate multiple cellular processes involved in cancer progression and that long-term treatment with β-blockers improves outcomes in several human cancers.^554^ In addition, evidence suggests that activated sympathetic nerves contribute importantly to changes in macrophage recruitment and differentiation that alter gene expression within the primary tumor.^555^

**Fig. 9:**
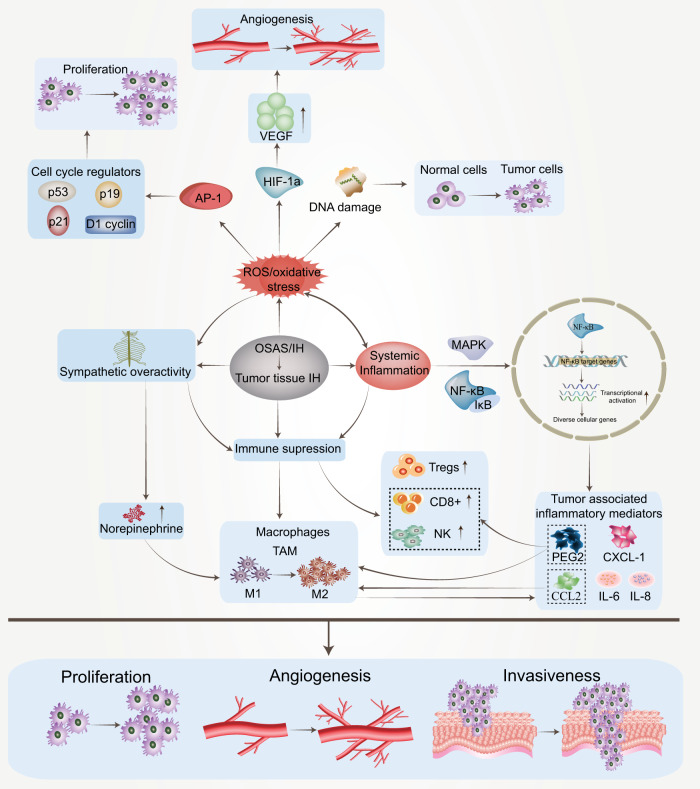
Potential mechanisms of the interaction between OSAS and cancer. IH could increase ROS levels in tumor tissues and further regulate cell cycle regulators through AP-1 to promote tumor proliferation. Elevated HIF-1α promotes the expression of VEGF and induces the growth of blood vessels in tumor tissues. The activation of NF-κB leads to the overexpression of tumor-related inflammatory mediators and tumor-related cellular immune dysfunction. In addition, enhanced sympathetic nerve activity releases norepinephrine, which can also change the tumor microenvironment and promote the occurrence of tumor cells. VEGF Vascular endothelial growth factor, CCL2 CC motif chemokine ligand 2, CXCL1 CXC motif chemokine ligand TAMs tumor-associated macrophages, M1 denotes antitumor phenotype macrophages, M2 denotes tumor-promoting phenotype macrophages

In conclusion, the available data suggest that OSAS might be an important risk factor for cancer development and aggressive cancer behavior. Data linking OSAS to the risk of neoplastic disease are scarce, but the above retrospective studies reveal the possibility of a close relationship (Fig. 9), which should stimulate more research on the effects of OSAS on carcinogenesis, tumor progression, and metastasis. In addition, there are currently no relevant studies reporting the complex links between sleep, adrenergic signaling, and cancer biology, suggesting a new direction for future research.

### OSAS and reproductive disorders

Emerging evidence suggests^556^ that IH associated with OSAS might contribute to reduced fertility and decreased testicle antioxidant capacity in male patients with this sleep-breathing disorder. In parallel, motility impairment of sperm and increased oxidative stress markers were observed in the testes of middle-aged and young mice subjected to IH, which resulted in reduced sperm motility. In addition, OSAS has been reported to cause alterations in male sexual function, and previous studies using IH in an animal model of OSAS showed that mice subjected to a chronic exposure protocol develop erectile dysfunction accompanied by decreased libido and impaired sexual capability.^557^ Multiple studies have confirmed that 10 to 60% of patients with OSAS may experience erectile dysfunction, and although erectile dysfunction is a frequently reported sexual dysfunction in males with OSAS,^558^ notably, OSAS also has a negative impact on sexual function in females.^559^ Interestingly, erectile dysfunction may be significantly improved after treatment with CPAP.^560^ As mentioned above, OSAS can cause reduced NO production and elevated levels of endothelin, leading to endothelial dysfunction, which results in increased vasoconstriction and impaired endothelial cell function. It has also been shown that IH increases oxidative stress in erectile tissue through the modulation of NADPH oxidase enzymes, leading to decreased NO production and subsequently to impaired penile tumescence.^557^ Another potential mechanism is the nocturnal suppression of testosterone release,^561^ as peak testosterone levels coincide with the onset of REM sleep, but patients with OSAS suffer from disrupted sleep and a reduction in the number and time of REM sleep episodes, which is associated with reduced circulating testosterone concentrations. In addition, hypo- and hypercapnia suppress the increase in blood testosterone levels during the night. The results from a large cohort study suggest that OSAS is associated with an increased risk of preeclampsia, eclampsia, and gestational diabetes, even after controlling for obesity.^562^ Another retrospective population-based dataset study found an increased risk of preeclampsia among pregnant women with OSAS, and these differences remained significant after controlling for obesity.^563^ Moreover, experimental studies in animals have found that pregnant rodents subjected to chronic hypoxia developed preeclampsia-like symptoms.^564^ IH-induced inflammation and oxidative stress are considered major contributors to end-organ damage in preeclamptic patients.^565^ OSAS-induced inflammation-related factors (TNF-α, IL-6, IL-8, and CRP) might act through synergistic pathways with the pathogenesis of preeclampsia.^566^ Evidence suggests that hypoxia-related signaling pathways in preeclampsia might be mediated by the immune system.^567^ At present, the mechanisms linking OSAS to preeclampsia are also not well defined, and we propose some plausible mechanisms, but few studies have investigated these potential pathways. This hypothesis remains to be further studied.

### OSAS and COVID-19

Coronavirus disease 2019 (COVID-19) is a severe respiratory-compromising disease caused by severe acute respiratory syndrome coronavirus 2 (SARS-CoV-2 virus) infection and is currently causing a pandemic. The link between OSAS and COVID-19 is biologically plausible. First, systemic chronic low-grade inflammation in patients with OSAS^407,408^ might contribute to a more severe immune response to COVID-19. Furthermore, OSAS could exacerbate the core symptoms of severe COVID-19, especially during the night, when oxygen saturation levels in OSAS become lower, resulting in more pronounced hypoxemia-, oxidative stress-, and hypoxia-related manifestations. Studies have shown that the risk of infection with COVID-19 was much higher in OSAS patients than in non-OSAS patients. Among patients with COVID-19 infection, OSAS was associated with an increased risk of hospitalization and could increase the risk of developing respiratory failure.^568^ OSAS is known to be strongly associated with male sex, obesity, and diabetes, all of which are well-recognized risk factors for severe COVID-19.^569^ It is inevitable that the limitations of these important confounders influence such conclusions. After addressing possible confounders, the most recent study found that OSAS was associated with a twofold increased risk of severe COVID-19, a finding that could not be explained by obesity or other comorbidities.^570^ These current findings strongly suggest that OSAS is an independent factor contributing to the risk of more severe COVID-19.^568,570,571^ The most damaging complication during COVID-19 is the cytokine storm involving IL, TNF-α, CRP, leptin, and ferritin. Similar inflammatory responses observed during OSAS have been described in detail previously. There is a close relationship between hypoxemia and cytokine storms, and hypoxia/reoxygenation in OSAS patients worsens hypoxemia, thereby aggravating cytokine storms.^572^ Moreover, HIF-1α and NF-κB, which are associated with OSAS, are fully involved in the triggering effect of hypoxemia on cytokine storm development.^573^ Notably, studies have established that SARS-CoV-2 enters host cells by binding to the angiotensin-converting enzyme-2 (ACE-2) receptor.^574,575^ ACE-2 is a noncanonical pathway of the renin-angiotensin system (RAS) pathway, and therefore, the RAS itself is involved in the pathogenesis of COVID-19.^576^ Interestingly, the increased expression of ACE-2 and dysregulation of the RAS in untreated OSAS patients due to IH have been shown,^577^ which could facilitate the entry of the SARS-CoV-2 virus into host cells, increase its viral load and infectivity, and ultimately lead to severe disease outcomes and mortality. In addition, patients with OSAS might have a higher susceptibility to the SARS-CoV-2 virus and might be more susceptible to the virus.

In conclusion, we propose that dysregulation of the RAS plays an important role in the pathogenesis of COVID-19 in OSAS patients and that IH might exacerbate cytokine storms in COVID-19, leading to acute respiratory distress syndrome and multiorgan failure. Data from the current study are very limited, and further studies are needed to better define the relationship between OSAS and COVID-19.

## Treatment

The treatment of OSAS aims to reduce symptoms, improve quality of life, reduce complications, and decrease mortality. Effective treatment of OSAS includes nonsurgical interventions (behavioral therapy, medical devices, and pharmacotherapy) (Table 7) and surgical procedures (Table 8). Behavioral therapy includes psychological education, cigarette smoking cessation, abstinence from alcohol and sedatives, aerobic exercise, weight loss, and avoiding the supine sleeping position. Behavioral therapy can address factors that may exacerbate OSAS. Regarding psychological education, doctors should communicate more with patients, patiently listen to their opinions and requirements, and explain in detail that OSAS is closely related to the occurrence of systemic diseases, which can help OSAS patients achieve a good psychological state to maintain a positive attitude toward the disease, which also contributes to improving patient compliance with subsequent treatment measures. Alcohol selectively decreases airway muscle tone and increases apnea frequency during sleep. In addition, alcohol also prolongs the duration of asphyxia by delaying arousal, and alcohol clearly interferes with the treatment of OSAS.^578–580^ A previous study showed that cigarette smoking might induce oropharyngeal narrowing and increase the severity of OSAS,^581^ and a recent meta-analysis found that secondhand smoke exposure is also significantly associated with OSAS.^582^ Cigarette smoking might increase the severity of OSAS by altering the sleep architecture, inducing upper airway inflammation, and interfering with upper airway neuromuscular function and arousal mechanisms.^583^ Weight loss may improve AHI in obese OSAS^584–586^ and should be recommended for all overweight or obese patients who are not suitable for other treatments. It could be used as the sole initial treatment for asymptomatic or minimally symptomatic patients. Recent studies have found that for OSAS patients with obesity, weight loss has been shown to be effective in reducing the tongue fat volume, which is directly related to a reduction in the AHI.^587^ In another randomized study, a lifestyle intervention that involved weight loss through diet and exercise resulted in a reduction of 10.2 kg and a reduction in the AHI of 9.7 events per hour in obese patients with type 2 diabetes mellitus and OSAS.^588^

**Table 7:** Primary nonsurgical interventions for obstructive sleep apnea syndrome (OSAS)

Treatment	Description	Indications and advantages	Downsides to treatment
Behavioral intervention			
Psychoeducation	Targeted mental health counseling should be carried out, communication with patients should be strengthened, and knowledge of the disease should be introduced to patients in plain language. Patients should be advised to stop drinking, smoking, and taking sedatives	It can help OSAS patients achieve a good psychological state in order to maintain a positive attitude toward the disease, which also contributes to improving patient compliance for subsequent treatment measures	
Weight loss^709–711^	Diet control, exercise therapy, and drug treatment	It is recommended for all overweight and obese patients diagnosed with OSAS; weight loss is beneficial for health and can improve cardiovascular and metabolic diseases and improve quality of life	It takes a long time; may not be effective for some patients; weight loss is hard to stick to
Exercise^589,712^	Choose suitable aerobic exercise, such as jogging, walking, swimming, and ball games	Contributes to weight control; it improves apnea independently of other mechanisms; reduces the risk of chronic diseases	May be difficult for patients with excessive body weight, muscle and joint damage, and severe cardiopulmonary dysfunction
Positional treatment^607^	Avoid sleeping in the supine position; tennis ball technique; chest position therapy device; neck position therapy device	Alternative treatments for patients with OSAS who are intolerant of PAP therapy; self-positioning has no cost; it is not expensive to wear	It is only applicable to patients with positional OSAS; shoulder problems or other physical disabilities can affect sleep in the side-lying position; adherence to treatment remains an issue
Pharmacologic Therapy^46^	Medical therapy focuses on improving upper airway muscle tone, ventilatory drive, or the arousal threshold	Complementary therapeutic approaches; to improve the treatment compliance of patients; availability of pharmacologic therapy opens up new directions for the pathophysiological phenotype of OSAS	There are currently no marker pharmacologic treatments available in OSAS; much effort has been made to pharmacologically improve airway patency, but a large number of studies have not been of very high quality; relevant experimental models of OSAS are lacking
Noninvasive medical treatment			
Positive airway pressure (PAP)^614,713^	PAP treatment delivers pressure to the upper airway by circulating compressed room air via a mask worn over the nose or the nose and mouth. There are three modes of PAP delivery: CPAP, BPAP, and APAP	First-line treatment of OSAS; it can effectively eliminate nocturnal snoring and other respiratory events, correct nocturnal hypoxemia, and improve sleepiness and blood pressure	Approximately one-third of patients have poor tolerability; may cause nasal injury, leading to local compression necrosis; not easily fixed
Mandibular advancement device (oral appliances)^626,714^	These devices are manufactured to accommodate the upper and lower teeth, are worn in the mouth, and during sleep, the lower jaw is kept in the anterior position	Patients with mild to moderate OSAS; PAP intolerant patients, PAP nonresponder patients, PAP treatment failure patients	There is a high cost and time required to build the equipment; temporomandibular joint discomfort, tooth pain, dryness of the mouth, or excessive saliva production

**Table 8:** Primary surgical treatment for obstructive sleep apnea syndrome (OSAS)

Treatment	Description	Indications and advantages	Downsides to treatment
Uvulopalatopharyngoplasty (UPPP)^636^	UPPP can expand the pharyngeal cavity and relieve the obstruction of the retropalatal plane by removing part of the hypertrophic soft palate tissue, palatal ptosis, the redundant soft tissue of the lateral pharyngeal wall, and hypertrophic palatine tonsil	Surgical methods are widely used; significantly improved symptoms in patients with OSAS	Surgical risks of the procedure; voice change, swallowing disorder, postoperative pain, and nasal regurgitation; recurrence occurs with weight gain; potential retroglossal collapse is not resolved
Maxillomandibular advancement^645,715^	Maxillary Le Fort I osteotomy, mandibular sagittal split ramus osteotomy, and infrahyoid muscle group transection of the hyoid bone suspension were performed	The forward movement of the upper and lower jaws dilates the upper airway, the tongue falls back, and the collapse of the airway is reduced; mandibular deficiency, severe OSAS with multiple obstructions	Surgical risks of the procedure; the operation is complicated and the recovery time is long; potential complications include poor cosmetic results and facial paresthesia
Nasal surgery^716^	It mainly includes septoplasty, turbinoplasty, and adenoidectomy	Nasal surgery is mainly used in CPAP-intolerant patients who have no response to medical treatment of nasal obstruction	Surgical risks of the procedure
Tracheostomy^634,717^	A tracheostomy is a surgical procedure that incises the anterior wall of the trachea at the cervical level to allow a new respiratory passage to be established	Used in emergency situations only; rarely, it is performed in cases where other treatments for severe OSAS are not feasible	An unacceptable cosmetic result; effects on verbal communication; easy intercurrent infection; need for long-term tracheotomy care
Bariatric surgery^718,719^	The most effective treatment for obesity; the three most common methods of weight loss in the United States are laparoscopic sleeve gastrectomy, Roux-en-Y gastric bypass, and laparoscopic adjustable gastric banding	Patients with OSAS (body mass index ≥35) who failed to achieve sufficient weight loss to achieve target health goals after behavioral therapy with or without medication	Contraindications include poor cardiac function, respiratory insufficiency, poor adherence to medication, and severe psychological disorders
Hypoglossal nerve stimulation^648,720^	The stimulation device is surgically implanted subcutaneously to stimulate the hypoglossal nerve to increase the tongue protrusion and expand the upper airway and improve airflow in and out	Patients unwilling or unable to tolerate PAP; endoscopy during induction of anesthesia revealed no centripetal collapse of the soft palate location; body mass index <32	Surgical site pain, infection, stiff tongue, pharyngeal pain, tongue muscle paralysis; expensive compared to alternative therapies

Exercise is often recommended in conjunction with weight loss. In fact, general exercise, when used as the sole intervention, modestly improved OSAS severity,^589^ and was independent of weight loss.^590–593^ In a study of a heart failure population, exercise alone reduced the AHI, and exercise with CPAP was associated with a significantly reduced AHI.^594^ Interestingly, in another randomized clinical trial of patients with OSAS, exercise was associated with a 24 to 34% reduction in OSAS severity, with no significant change in body weight.^591–593^ The mechanism of this weight-independent improvement in OSAS is unclear. Redistribution of fat, decreased nocturnal leg fluid absorption, improved sleep quality, and increased pharyngeal muscle strength are thought to be underlying mechanisms of action. In another study of the association between exercise volume and OSAS prevalence, compared with individuals who did not exercise vigorously, those who exercised 1 to 2 h weekly, 3 to 6 h weekly, and at least 7 h weekly had odds ratios for moderate-to-severe OSAS of 0.62, 0.39, and 0.31, respectively.^590^

Positional OSAS was first defined by the Cartwright criteria,^595^ that is, the AHI during nonsupine sleep was at least 50% lower than that during supine sleep. Since then, its definition has been reiterated several times.^596^ A recent study applying Cartwright’s definition of positional OSAS found that 35.3% of a large number of patients with severe OSAS had positional sleep apnea.^597^ Alternatively, several studies have estimated that approximately half of OSAS cases appear or worsen only during supine sleep.^598–600^ There are multiple anatomical and physiological changes in the respiratory system capable of increasing the propensity for sleep-disordered breathing when switching from the nonsupine to the supine position. These include an increase in the loop gain,^601^ a reduction in airway diameter^602,603^ and a reduction in functional residual capacity.^604^ Traditional positional therapy is a variation of the “tennis ball technique” (TBT) and involves strapping a bulky object to the patient’s back to discourage supine sleep.^605^ This technique is effective in reducing supine sleep duration and is simple and affordable, but it is often uncomfortable for patients and therefore has poor long-term adherence. One study found that only 6% of patients adhered to the TBT at 2.5 years, which was stopped mainly due to discomfort.^606^ Although there are no standardized approaches to positional therapy and prospective data on its efficacy are lacking, for patients with positional OSAS, restricting sleep to the lateral or prone position may be an effective treatment.^607,608^

In 1981, Collin Sullivan proposed positive airway pressure (PAP) therapy^609^ as the primary treatment for patients with symptomatic OSAS of any severity.^610^ PAP treatment delivers pressure to the upper airway by circulating compressed room air via a mask worn over the nose or the nose and mouth. The elevated air pressure acts as a splint to prevent upper airway collapse during inspiration and improve oxygenation, thereby enabling normal breathing.^609,611^ There are many other different PAP options available, depending on the mode of positive pressure delivery and the setup.^612^ CPAP devices apply a fixed positive pressure, requiring pressure titration in the laboratory to determine the optimal treatment pressure. In patients with OSAS who cannot tolerate CPAP fixed pressure, autotitrating positive airway pressure (APAP) devices could be used. APAP can monitor airflow and adjust the delivered pressure in response to flow rate changes, airway resistance, and pressure changes,^613^ which contributes to initiating PAP therapy without laboratory titration, reduces costs, and increases convenience, and there is no significant difference in the efficacy or treatment compliance between laboratory titration and automatic titration.^614^ However, APAP devices may not be appropriate for patients with CSA or nocturnal hypoxemia due to causes other than sleep apnea. Bilevel positive airway pressure (BPAP) devices deliver higher pressures during inhalation than exhalation and may be considered to improve hypercapnia better in OSAS with other comorbidities (obesity hypoventilation syndrome) but are neither more effective nor more tolerated than CPAP or APAP devices. When an OSAS patient wears the device regularly during sleep, PAP normalizes the AHI to avoid apnea events in more than 90% of patients.^614–616^ Treatment effectiveness was dependent on adherence to device use, with longer nightly wear associated with greater improvement in symptoms^617^ and greater blood pressure reduction.^618^ Although adherence was arbitrary, adequate adherence was generally defined as use for 4 or more hours nightly for at least five nights per week.^619^ However, many patients with OSAS cannot tolerate PAP devices, resulting in poor compliance.^620^ Unfortunately, reported nonadherence rates range from 46 to 83%.^621^ In addition, many studies have also reported low adherence and irregular use status of CPAP.^622–624^ Measures to improve PAP adherence include informing of OSAS risks and expected benefits of PAP treatment, monitoring PAP use, and enhancing support for technical issues. Each of these measures increased PAP compliance by more than 30 min per night.^625^

Oral appliances are effective treatment options, especially for patients with mild to moderate OSAS.^626,627^ In addition, this option is also indicated for patients who are intolerant of CPAP, nonresponders to CPAP, CPAP treatment failure, or patients with more severe OSAS who prefer alternative treatments.^586,628^ The most common designs are mandibular advancement devices, palate lift devices, and tongue retention devices.^629^ Mandibular advancement devices have become a popular means of oral appliance treatment due to the poor adherence of palate lift devices and tongue retention devices.^630^ These devices are constructed of steel plates that fit into the upper and lower teeth. These combined plates can be adjusted to allow the mandible to advance relative to the maxilla, with the aim of enlarging the oropharynx and velopharynx during sleep and activating stretch receptors to reduce airway collapse and improve upper airway patency.^631,632^ A multicenter study of more than 400 patients treated with mandibular advancement devices found that the AHI of OSAS patients became normal (AHI < 5) in 37% of patients, decreased to <10 in 52%, and was more than halved in 64%.^633^ A recent meta-analysis of randomized clinical trials found that these devices were strongly associated with improvements in the AHI (mean reduction in the AHI of 13.6 events/hour).^626^

Tracheotomy was used to treat severe OSAS before the advent of PAP therapy, with the advantage of bypassing airway obstruction and significantly improving OSAS, but it is now rarely used in the management of OSAS.^634^ The most common surgical treatment for OSAS is uvulopalatopharyngoplasty (UPPP), which expands the oropharyngeal airway and reduces pharyngeal collapse by altering the upper airway soft tissues, including the lateral pharyngeal walls, tongue base, and palate.^635,636^ According to the available reports, the AHI and lowest oxygen saturation of the blood are significantly improved after surgery, the oropharyngeal cavity diameter is significantly increased, and the surgical treatment rate of UPPP is approximately 33%.^637,638^ Multiple randomized trials have found significant reductions in the AHI with UPPP compared with observation controls.^639,640^ In these larger trials (32 surgery patients and 33 control patients), surgery was strongly associated with a mean decrease in the AHI from 53.3 to 21.1 beats per hour, whereas no significant change was observed in the control group.^639^ However, in patients with severe OSAS, its effect on AHI is limited, and long-term adverse effects have been reported.^641–643^ The limitations of UPPP include its failure to improve the lateral dimensions of the upper airway, to address retroglossal collapse, or to address the reduction in upper airway dilator muscle tone.^643^ Therefore, UPPP combined with other surgical treatments is necessary. Liu et al. found that UPPP combined with tongue base radiofrequency ablation increased the total effective rate of OSAS to 71.9%.^644^ Therefore, patients with retropalatal collapse are more suitable for UPPP, although this is difficult to diagnose definitively.^642^ Surgical modification of the facial bone structure can also be used to treat OSAS. The most studied procedure is maxillomandibular advancement, which combines a standard Le Fort I osteotomy with a sagittal split mandibular osteotomy to facilitate maxillary and mandibular advancement and to fix the facial skeleton by approximately 10 mm forward. It achieves upper airway dilation by physically expanding the skeletal frame of the face. A recent meta-analysis of individual data from 45 studies, including 455 patients/interventions showed that maxillomandibular advancement surgery was associated with an average 80% reduction in the AHI, consistent with a mean change of -47.8 (25.0) events/hour.^645^

Hypoglossal nerve stimulation is an advanced surgical treatment that can improve the tone of pharyngeal dilator muscles during sleep.^646^ At present, the most widely used technique and most used commercial implantation system places the stimulating electrode on the medial branch of the right hypoglossal nerve to enhance the ipsilateral tongue process. The respiration-sensing sensor is placed between the internal and external intercostal muscles to detect inspiratory power, and an implantable pulse generator is implanted in the chest wall to trigger hypoglossal nerve electrodes in response to respiratory effort.^647,648^ Adult patients with moderate-to-severe OSAS who failed or could not tolerate noninvasive treatment were recruited in a multicenter prospective single-group trial. Patients with OSAS had an AHI of 20 to 50 and a BMI of ≤32. In addition, exclusion criteria included CSA, positional OSAS, severe cardiopulmonary or neuromuscular disease, or concentric collapse of the retropalatal airway on drug-induced sleep endoscopy. When assessed after 12 months, this surgical modality reduced the median AHI from 29.3/h to 9/h.^649^ Treatment with hypoglossal nerve stimulation was associated with quality of life and improvements in sleepiness after 5 years, with a 63% remission rate.^650^ There were no serious adverse events. Thus, hypoglossal nerve stimulation is a surgical treatment with sustained benefits. Recently, a novel device known as the GENIO system has been developed to provide bilateral hypoglossal nerve stimulation for moderate-to-severe OSAS, resulting in a 45% decrease in the AHI,^651^ and transcutaneous stimulation is also under investigation.^652^ Although treatment with hypoglossal nerve stimulation appears to be effective and well tolerated, it is invasive and more costly than oral appliances and PAP.

Currently, there are no effective drugs available to treat OSAS. However, along with the development of modalities to address the nonanatomical pathogenesis of OSAS (pharyngeal critical closing pressure, muscle responsiveness, loop gain, nocturnal rostral fluid shift, and arousal threshold), it is helpful to guide the pharmacological development of novel OSAS targeted therapies (Table 9). Usually, hypnotic agents are contraindicated in OSAS due to concerns about upper airway muscle relaxation. Nevertheless, recent studies have shown that drugs such as eszopiclone could increase the arousal threshold and reduce the AHI without hypoxemia, which can be used as an adjuvant treatment for OSAS patients with good upper airway muscle activity and a low arousal threshold.^653^ Furthermore, standard doses of zolpidem affected respiratory arousal thresholds to varying degrees and did not interfere with pharyngeal muscle activity during sleep.^654^ Acetazolamide, a carbonic anhydrase inhibitor with diuretic properties that stimulates respiratory excitation through metabolic acidosis, has been shown to decrease the loop gain associated with OSAS, thereby improving ventilatory stability.^655–657^ In a study involving 13 patients with OSAS, acetazolamide (500 mg twice daily) for 1 week resulted in a 40% reduction in loop gain and a 50% reduction in the AHI.^655^ Another study involving 13 men with moderate-to-severe OSAS randomized participants to acetazolamide alone, CPAP alone, and acetazolamide + CPAP. Two weeks later, the AHI had decreased in all three groups, with the acetazolamide + CPAP group showing the greatest AHI reduction.^658^ A previous study showed that aminopyridine (a potassium channel blocker) is able to improve genioglossus activity during REM sleep.^659^ It is well known that potassium conductance mediates the reduction in motor neuron excitability by neuromodulators. Blocking some potassium channels in the hypoglossal motor pool could significantly enhance the activity of the genioglossus in sleep, which provides a novel direction for research on OSAS drug treatment.^660^ Interestingly, topical administration of potassium channel blockers increased upper airway reflex activity in animals and prevented negative pressure-induced upper airway collapse.^661^ Further studies are needed to clarify the role of potassium channel blockers in OSAS in humans. For OSAS patients with weaker muscle function, the tricyclic antidepressant desipramine reduces the severity of OSAS by preventing the sleep-induced decrease in genioglossus activity, thereby improving upper airway collapse.^662^ A recent study evaluated the AHI in patients with significant OSAS with the combination of atomoxetine (a norepinephrine reuptake inhibitor) and oxybutynin (an antimuscarinic agent).^663^

**Table 9:** Targeted pharmacotherapy to treat obstructive sleep apnea syndrome (OSAS)

Class	Pharmacotherapeutic agents	Reference	Mechanism of action
Anatomical impairment	Liraglutide	Blackman et al. (2016)^721^	Reduce body weight, leading to a decrease in upper airway fat (due to obesity) and thus reduce narrowing and/or the propensity for closure during sleep, which may decrease Pcrit in susceptible individuals
	Spironolactone and furosemide	Blackman et al. (2018)^722^	Reduce fluid retention, thereby reducing nighttime fluid transfer from the limbs to the neck
	Nasal decongestants (Mometasone alone)	Acar et al. (2013)^723^	Reducing nasal resistance can induce pharyngeal dilatation by decreasing the negative suction pressure downstream in the velo- and oropharynx
	Fluticasone	Kiely et al. (2004)^724^	
	Nasal steroid dexamethasone with the decongestant tramazoline	Koutsourelakis et al. (2013)^725^	
Low arousal threshold	Triazolam	Berry et al. (1995)^726^	Raising the arousal threshold might have the potential to buy time for the upper airway muscle recruitment and the stabilization of airway patency; zolpidem, diphenhydramine, and lorazepam all increased arousal threshold; lorazepam and zolpidem increased genioglossus activity before arousal in response to hypercapnia
	Lorazepam Zolpidem Diphenhydramine Eszopiclone	Carberry et al. (2017)^654^ Carter et al. (2016)^727^ Eckert et al. (2011)^653^ Rosenberg et al. (2006)^728^ Carberry et al. (2017)^654^ Park et al. (2008)^729^	
	Sodium oxybate	George et al. (2011)^730^	Sodium oxybate reduces the severity of sleep apnea by increasing deep sleep time and increasing the arousal threshold
	Trazodone	Eckert et al. (2014)^731^ Smales et al. (2015)^732^	Trazodone can increase the arousal threshold in response to hypercapnia and allow tolerance to higher CO_2_ levels without arousal, thus stabilizing sleep
High loop gain	Carbonic anhydrase inhibitor: Zonisamide and Acetazolamide	Eskandari et al. (2014)^733^ Eskandari et al. (2018)^658^ Edwards et al. (2013)^734^ Edwards et al. (2012)^655^ Schmickl et al. (2020)^735^ Schmickl et al. (2021)^656^ Tojima et al. (1988)^736^	Agents targeting loop gain reduce the PCO_2_ reserve by producing transient metabolic acidosis and relative hyperventilation, thus widening the difference between eupneic paCO_2_ and the apneic threshold, effectively reducing loop gain by reducing plant gain, stabilizing ventilator drive leading to respiratory tract opening and decreasing obstructive events
	Oxygen therapy	Sands et al. (2018)^737^ Wellman et al. (2008)^738^ Pokorski et al. (2000)^739^ Joosten et al. (2021)^740^ Wang et al. (2018)^741^	Oxygen therapy can reduce the circulation gain by quieting the chemosensory output of an overly sensitive chemoreflex system, which converts the perceived change in gas tension into a smaller change in the ventilatory drive.
	Carbon dioxide Rebreathing	Dempsey et al. (2004)^742^ Messineo et al. (2018)^743^ Xie et al. (2013)^744^	CO_2_ is added during hyperpnea to prevent transient hypocapnia to stabilize periodic respiratory abnormalities. In patients with high loop gain, CO_2_ rebreathing seems to be a promising treatment
Poor muscle responsiveness	Noradrenergic mechanisms: Desipramine, Protriptyline, Atomoxetine, and Antimuscarinic oxybutynin	Taranto-Montemurro et al. (2016)^662^ Taranto-Montemurro et al. (2016)^745^ Hanzel et al. (1991)^746^ Smith et al. (1983)^747^ Bart Sangal et al. (2008)^748^	By identifying the receptor targets that stimulate the upper airway muscles, we can manipulate the airway muscle tone to prevent upper airway muscle relaxation, restore pharyngeal muscle activity, and then restore upper airway patency through reflexive recruitment; desipramine could increase genioglossus activity and reduce upper airway collapse during sleep in humans
	Serotonergic mechanisms: Ondansetron, Buspirone, Mirtazapine, Paroxetine, Fluoxetine, and l-Tryptophan	Veasey et al. (2001)^749^ Mendelson et al. (1991)^750^ Carley et al. (2007)^751^ Berry et al. (1999)^752^ Hanzel et al. (1991)^746^ Schmidt et al. (1983)^753^	Serotonergic drive is attenuated centrally from wakefulness to NREM sleep and reaches a minimum during REM sleep, resulting in a relative reduction in ventilatory drive. Central administration of serotonin mediates respiratory excitation through 5-HT2a/c receptors on upper airway motoneurons and 5-HT1a receptors on respiratory neurons. Serotonin has different effects on central and peripheral respiration, but 5-HT3 antagonists and 5-HT1a agonists consistently improve respiration
	K^+^ channel blockers: 4-aminopyridine, Tetraethylammonium, and Doxapram	Grace et al. (2013)^754^ Suratt et al. (1986)^755^	Blocking potassium channels promotes membrane depolarization and cellular excitability, which leads to increased genioglossus activity during REM and NREM sleep; cannabinoids improve respiratory stability by attenuating the feedback of the vagus nerve to the medulla to help stabilize breathing and activate pharyngeal muscles
	Cannabinoids	Guo et al. (2004)^756^ Prasad et al. (2013)^757^	
	Nicotine	Gothe et al. (1985)^758^	
Other pharmacotherapeutic agents involved in OSAS			
Forskolin		Aoki et al. (1985)^759^	During wakefulness and non-REM sleep, forskolin increases cAMP at the hypoglossal motor nucleus, which in turn increases the activity of the pharyngeal muscle
Xanthines		Lagercrantz et al. (1985)^760^	Increase ventilation by antagonizing adenosine in the central nervous system and increasing diaphragm contractility

## Conclusion

The past two decades have seen unprecedented growth in sleep medicine, mostly owing to the growing awareness of OSAS and its profound impact on patient’s quality of life. As described above, epidemiological data and evidence from clinical trials, animal studies, and in vitro experiments indicate that IH caused by OSAS could lead to the activation of different signaling pathways and is closely related to the damage to multiple tissues and organs, in which oxidative stress, inflammation, and sympathetic activation are essential components of OSAS-related diseases, and IH plays an important role in the pathogenesis, development, and prognosis of multiple diseases. More in vitro and animal studies at the cellular level (different cell types) are needed in future studies to uncover new underlying mechanisms of IH and to predict new IH-related diseases.
